# Understanding the impact of climate change on mental health from a Canadian perspective: a scoping review

**DOI:** 10.24095/hpcdp.46.7/8.01

**Published:** 2026-07

**Authors:** Sarah V.C. Lawrason, Salah U. Khan, Amy Farrow, Mathilde Lavigne-Robichaud, Cailey Ellis, Nana Amankwah, Rojiemiahd Edjoc, Seana N. Semchishen

**Affiliations:** 1 Public Health Agency of Canada, Ottawa, Ontario, Canada

**Keywords:** climate change, mental health, Canada, scoping review

## Abstract

**Introduction:**

Climate change poses a growing threat to health in Canada through both extreme weather and climate events (e.g. wildfires, floods, extreme heat) and gradual environmental changes (e.g. shifting seasonal patterns, ecosystem degradation). While physical health impacts have been well documented, mental health consequences have only recently gained attention. This scoping review synthesizes research examining how climate-related weather events influence mental health outcomes across diverse populations and regions in Canada.

**Methods:**

Following Arksey and O’Malley’s five-stage scoping review framework, 72 studies published between 1990 and 2024 were analyzed. Mental health impacts were categorized by type of weather and climate event and exposure factors, population subgroup, and outcomes according to length of impact.

**Results:**

Depression, anxiety, and posttraumatic stress disorder (PTSD) were the most commonly reported conditions, particularly following wildfires and floods. Climate-related exposures such as property loss, displacement, and disruption of livelihoods were identified as key factors leading to psychological distress. Vulnerable populations—including Indigenous peoples, youth, women, low-income groups, and individuals with pre-existing conditions—were disproportionately affected. Emerging concerns such as eco-anxiety and grief tied to environmental change were frequently reported, especially in qualitative studies. Long-term effects of climate change on mental health were evident in more than half of the included studies, highlighting the enduring nature of these impacts.

**Conclusion:**

Despite growing evidence, gaps remain in understanding region-specific exposures, gradual climate stressors, and protective interventions. This review emphasizes the need for targeted research, policy development, and mental health programming to address climate-related disparities and enhance resilience across Canada’s diverse populations.

## Highlights

• We conducted a scoping review to summarize research on climate change and mental health in Canada.

• Wildfires, floods and extreme heat are linked to more depression, anxiety and PTSD.

• Groups facing existing challenges—such as Indigenous peoples, youth, women and those with low income—are especially at risk.

• Eco-anxiety and grief over environmental loss are increasing, and more support is needed for vulnerable communities.

## Introduction

Climate change in Canada is driving both extreme, acute weather and climate events (specific weather events or hazards, e.g. flooding, tornados, wildfires) and gradual environmental changes (longer-term shifts in climate patterns – e.g. rising sea levels, increasing temperatures). Climate change has a wide-ranging impact on society, such as undermining ecosystems, disrupting the stability of food and water resources, and the erosion of habitats and cultural heritage. Several regions across Canada are experiencing greater impacts of climate change, with one study showing that the costs of weather-related insured losses from 2010 to 2019 were twice as high as those reported from 1983 to 2009.^1^

Research has established the impact of climate change on physical health and safety (e.g. increased temperatures are associated with higher cardiovascular-related hospitalizations).^2^ However, recent studies emphasize the negative impact of climate change on mental health and well-being.^3^^,^^4^ For instance, the World Health Organization 2022 policy brief highlighted how climate-related hazards (e.g. storms), long-term environmental risks (e.g. contaminated water), and individual vulnerabilities (e.g. pre-existing health conditions) can synergistically impact mental health.^5^ Furthermore, the Intergovernmental Panel on Climate Change report underscores the growing risks climate change poses for mental health and well-being, and calls for urgent adaptation strategies to mitigate these risks.^6^ If risks are appropriately mitigated, then outcomes such as displacement, cultural disruption, and social inequity can be avoided or at least reduced in severity.

Climate change can affect mental health through direct pathways, such as the immediate psychological impact of extreme events, where there is more frequent exposure to physical danger, chronic stress, and elevated rates of violence and aggression.^7^ Indirect pathways include the gradual effects of long-term exposure to environmental and social degradation on communities (e.g. damage to homes and infrastructure, more frequent physical health impacts and damage to livelihoods and socialization).^7^ Research, including a global scoping review, underscores that events such as wildfires and floods are particularly detrimental to mental health, especially among at-risk groups such as children, Indigenous peoples, women, low to middle-income persons, and those with pre-existing illnesses.^8^^,^^9^

Systemic changes in Canada’s economy and ecosystems are expected to take place due to climate change, and population mental health can worsen due to increased worry, trauma, depression, and suicidal behaviours.^10^^,^^11^ Health Canada recently published a report on the changing climate, including a comprehensive chapter dedicated to mental health that summarized the increasing burden of mental illness in Canada because of climate change.^10^ However, greater understanding is needed on the specific mechanisms that contribute to the impact of climate change on mental health in Canada, so that policy and program interventions can be developed appropriately. For example, Canada experiences unique extreme and gradual environmental events across diverse regions, including droughts, floods, wildfires, and extreme heat events. There is a need to assess how extreme weather and climate events and gradual climate change in the Canadian context can influence mental health.

The purpose of this scoping review was to understand how different weather and climate events and changes affect population mental health outcomes. We aimed to identify risks for the Canadian population, and how these risks may disproportionately affect equity-owed populations. To achieve this goal, we 1) synthesized existing literature on weather and climate events and mental health outcomes; 2) assessed factors of exposures that lead to worsened mental health; and 3) report on the different lengths of impact of mental health outcomes. The objective of this review was not to re-establish the somewhat intuitive relationship between mental health and climate change, but rather to clarify how climate events affect mental health across different populations and time within a Canadian context. By establishing extreme versus gradual or perceived events, we highlight their impact on a differential set of mental health outcomes. In doing so, this reframes climate-related mental health impacts from a singular outcome to a set of context-specific, equity-sensitive processes.

## Methods

We performed a scoping review to summarize information on negative mental health outcomes in relation to climate change-associated weather events in Canada. The five-stage framework for scoping reviews developed by Arksey and O’Malley^12^ informed the review strategy and approach: 1) identify the research question; 2) identify relevant studies; 3) select studies; 4) chart the data; and 5) summarize the results. Because of the broadness of the topic of climate change, perceived heterogeneity between studies, and a lack of rich descriptive and quantitative data, we did not perform a meta-analysis. The research proposal was approved by the Science Review Committee within the Public Health Agency of Canada, and the protocol is not publicly available. The protocol and article retrieval was developed a priori.

### Stage 1: Identify the research question

Through consultation between researchers and policy analysts at the Public Health Agency of Canada, the primary research question was: What is known from the literature about the relationship between climate change and mental health among Canadians? We were interested in both extreme and gradual categories of climate change (including direct exposure to extreme events and general concern about climate change), pathological and non-pathological mental states (e.g. diagnoses of depression and eco-anxiety, respectively), and we were interested in all populations.

### Stage 2: Identify relevant studies

The search strategy was developed by the study authors in collaboration with a librarian at the Public Health Agency of Canada. The search was informed by previous reviews of climate change and mental health.^8^^,^^9^ Search terms were related to the following categories: climate change (and weather events, e.g. wildfires, greenhouse gases), AND negative mental health (e.g. mood disorders, eco-anxiety, depression). The full strategy can be found in Appendix A.

The librarian conducted searches for articles published between 1990 and 2022 among the following databases: Medline, Embase, and PsycINFO databases with a country-specific search published between 1990 up until the search date (18 January 2023). An updated search was performed on 19 December 2024, to capture articles published from 2022 to 2024. De-duplication was conducted in Covidence. Hand-searching of reference lists of included studies was conducted to search for additional relevant articles.

In addition, a grey literature search was conducted. The librarian helped to suggest databases and search strategy. The following databases were searched using the same key terms as above: Canada’s Drug Agency – Grey Matters, Theses Canada, Web of Science Core Collection (limited to Conference Proceedings Citation Index), BIOSIS Citation Index, Scopus, and HealthADAPT. These results were manually screened for duplication.

### Stage 3: Select studies

This review included both 1) title and abstract; and 2) full-text screening. Three independent reviewers tested the reliability of the article selection tool using a sample of abstracts prior to the review. The articles were independently screened by title/abstract against the inclusion criteria, and those included articles were then screened again using the full text. Reviewers consulted with other authors when issues arose. The reviewers regularly met to reconcile extractions and discussed any discrepancies. Neither reviewer was blinded to the study’s title, author, or institution.

Articles were included in the review if they recruited Canadians of any age, mentioned a presence of any climate change–associated weather event(s), any mental health outcome, and the articles were primary research studies. The inclusion criteria were meant to be broad to be able to map the literature appropriately, and to identify gaps in the empirical literature in the Canadian context. The search was limited to primary research to maintain consistency in study design and outcome reporting. Articles that did not meet all of the inclusion criteria or were not published in the English or French language (official languages of Canada) were excluded. Articles that included populations combining Canada with other countries were also excluded. Figure 1 displays the PRISMA flow chart (Moher et al., 2009) presenting the search strategy process.

**Figure 1:**
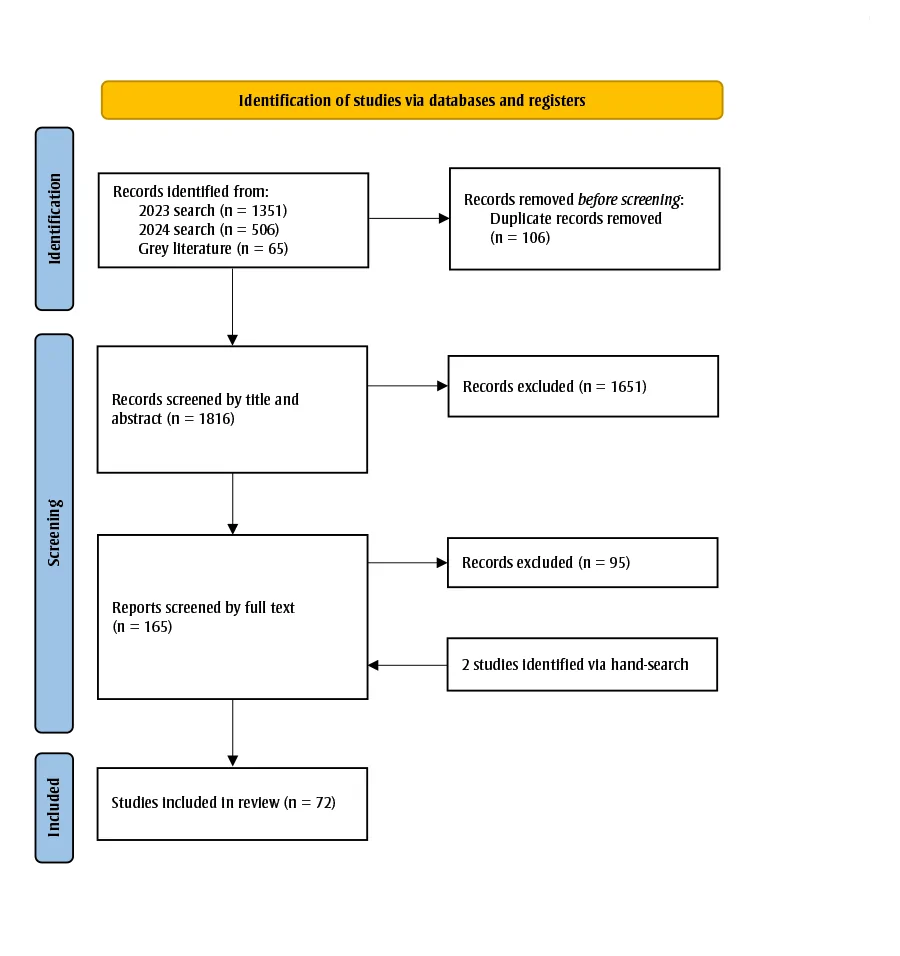
A PRSIMA flow diagram of the study identification and selection process to assess mental health impacts due to climate change in Canada, 1990 to 2023 **Source:** Page MJ, McKenzie JE, Bossuyt PM, et al. The PRISMA 2020 statement: an updated guideline for reporting systematic reviews. BMJ. 2021;372:n71. https://doi.org/10.1136/bmj.n71

### Stage 4: Chart the data

Two independent reviewers completed the data extraction process. The authors discussed the tool before extracting the data to ensure clarity and consistency. The following information was extracted from each article: author(s); year of publication; title; study design; sample size; population and subgroups; location; type of data collected; climate event; mental health outcomes; healthy equity information; and key conclusions. Inconsistencies between authors were reconciled through discussion. The authors were not blinded to data extraction and charting.

### Stage 5: Collate, summarize and report the results

The results were summed up according to type of event; mental health outcomes; duration of impact; and potential factors influencing the pathway between exposure and outcome. Climate events were categorized according to the type of extreme or gradual event (e.g., extreme events including but not limited to flooding, wildfire, or gradual events such as perceived general climate change). Mental health outcomes were categorized according to the outcomes reported in articles (e.g. anxiety, depression, posttraumatic stress disorder (PTSD)). Mental health impacts were defined as experiencing symptoms or diagnoses relating to mental or behavioural challenges.^7^ The duration of mental health impact was categorized from the beginning of the exposure to the outcome measurement according to the review by Berry et al.,^7^ including short (≤ 2 months), intermediate (> 2 to ≤ 11 months), and long (≥ 1 year). Prevalence estimates of mental health variables had a definition which included either a diagnosis by a health care provider, self-report of a pre-existing diagnosis, or use of a validated scale in a questionnaire. Qualitative studies that mentioned the presence of mental health outcomes in interviews did not provide a mental health assessment or diagnosis; however, themes relating to mental health were still extracted from these studies and informed the exposure factors analysis. Terms relating to exposures and mental health outcomes that appeared the most often together were used to construct the climate change and mental health impact factors from the narrative text. Importantly, this is by no means defining causal pathways—it is rather to be taken as hypotheses or a potential framework. We performed descriptive analysis and narrative synthesis of the point estimates of mental health outcomes, geographical distribution, climate and extreme weather and climate event types.

## Results

### Articles retrieved

The search in 2023 retrieved 1351 articles, the search in 2024 retrieved an additional 506 articles, and the grey literature search retrieved 65 results for a total of 1816 articles after removing duplicates (see Figure 1). After title/abstract and full-text screening, 72 articles met the inclusion criteria (see Appendix B for a list of included articles). One article was retrieved through the grey literature search (through HealthADAPT). Of these articles, 49 were quantitative, 21 were qualitative, and 2 were mixed methods. Characteristics of articles can be found in Table 1 and data extracted from each article are presented in Table 2.

**Table 1:** Included articles in the scoping review on mental health and climate change research in Canada from 1990 to 2023

**Author**	**Title**	**Province and area**	**Climate event**	**Mental health assessment**
Adu 2022	Five years after the Fort McMurray wildfire: prevalence and correlates of low resilience	Fort McMurray, Alberta	Specific event: Wildfire	Questionnaires: PHQ-9, GAD-7, PCL-C, BRS
Adu 2024	Exploring the prevalence and predictors of low resilience and likely PTSD in residents of two provinces in Canada during the 2023 wildfires	Alberta and Nova Scotia	Specific event: Wildfire	Questionnaire: PCL-C
B. Agyapong 2022a	Mental health impacts of wildfire, flooding and COVID-19 on Fort McMurray school board staff and other employees: a comparative study	Fort McMurray, Alberta	Specific event: Wildfire, flood	Questionnaires: Ad-hoc self-report survey, GAD-7, PHQ-9, PCL-C, BRS
B. Agyapong 2022b	Cumulative trauma from multiple natural disasters increases mental health burden on residents of Fort McMurray	Fort McMurray, Alberta	Specific event: Wildfire, flood	Questionnaires: Ad-hoc self-report survey, GAD-7, PHQ-9, PCL-C, BRS
B. Agyapong 2022c	Self-reported alcohol abuse and the desires to receive mental health counselling predict suicidal thoughts/thoughts of self-harm among female residents of Fort McMurray	Fort McMurray, Alberta	Specific event: Wildfire, flood	Questionnaires: Ad-hoc self-report survey, PHQ-9, GAD-7, PCL-C, BRS
V. Agyapong 2018	Prevalence rates and predictors of generalized anxiety disorder symptoms in residents of Fort McMurray six months after a wildfire	Fort McMurray, Alberta	Specific event: Wildfire	Questionnaires: GAD-7, AUDIT, DUDIT
V. Agyapong 2020	Long-term mental health effects of a devastating wildfire are amplified by sociodemographic and clinical antecedents in elementary and high school staff	Fort McMurray, Alberta	Specific event: Wildfire	Questionnaires: Ad-hoc self-report survey, PHQ-9, GAD-7, PCL-5, AUDIT, DUDIT
V. Agyapong 2021	Prevalence rates and correlates of likely post-traumatic stress disorder in residents of Fort McMurray six months after a wildfire	Fort McMurray, Alberta	Specific event: Wildfire	Questionnaires: PCL-5, AUDIT, DUDIT
Auger 2000	L’état de stress post-traumatique: l’après-déluge au Saguenay (Posttraumatic stress disorder: the aftermath of the flood in Saguenay)	Chicoutimi, Quebec	Specific event: Flooding	Questionnaires: PTSD Reaction Index, SRQ
Beaumier 2010	Food insecurity among Inuit women exacerbated by socioeconomic stresses and climate change	Igloolik, Nunavut	Perceived climate change: Changing environmental conditions (later and longer ice free-up, thinner ice, increasing temperatures, changing weather patterns)	Qualitative: Semi-structured interviews and focus groups with Inuit women, and key informant interviews with health professionals
Belanger 2014	Perceived adverse health effects of heat and their determinants in deprived neighbourhoods: a cross-sectional survey of nine cities in Canada	Quebec (Nine unspecific cities)	Specific event: Extreme heat	Qualitative: Interviews Questionnaire: CCHS
Belleville 2019	Post-traumatic stress among evacuees from the 2016 Fort McMurray wildfires: exploration of psychological and sleep symptoms three months after the evacuation	Fort McMurray, Alberta	Specific event: Wildfire	Questionnaires: PCL-5, PHQ-9, ISI, PTCI, WCQ, PSQI, PSQI-A Diagnostic interviews: CAPS, MINI
Belleville 2021	Psychological symptoms among evacuees from the 2016 Fort McMurray wildfires: a population-based survey one year later	Fort McMurray, Alberta	Specific event: Wildfire	Questionnaires: PCL-5, ISI, PHQ-9, GAD-7, CAGE
Brown 2019a	Significant PTSD and other mental health effects present 18 months after the Fort McMurray wildfire: findings from 3070 grade 7–12 students	Fort McMurray, Alberta	Specific event: Wildfire	Questionnaires: CPSS, HADS, PHQ-A, CRAFFT, Tobacco Use Questionnaire, Rosenberg Self-Esteem Scale, KIDSCREEN Questionnaire, CYRM
Brown 2019b	After the Fort McMurray wildfire there are significant increases in mental health symptoms in grade 7–12 students compared to controls	Fort McMurray, Alberta	Specific event: Wildfire	Questionnaires: HADS, PHQ-A, CRAFFT, Tobacco Use Questionnaire, Rosenberg Self-Esteem Scale, KIDSCREEN Questionnaire
Brown 2021	Mental health symptoms unexpectedly increased in students aged 11–19 years during the 3.5 years after the 2016 Fort McMurray wildfire: findings from 9376 survey responses	Fort McMurray, Alberta	Specific event: Wildfire	Questionnaires: CPSS-19, PHQ-A-11, HADS-9, CRAFFT, Tobacco Use Questionnaire, Rosenberg Self-Esteem Scale, KIDSCREEN Questionnaire, CYRM
Bunce 2016	Vulnerability and adaptive capacity of Inuit women to climate change: a case study from Iqaluit, Nunavut	Iqaluit, Nunavut	Perceived climate change: Increasing temperatures, changes to snow and ice cover (e.g. thinner ice), more frequent rain-on-snow events	Qualitative: Interviews, focus groups
Chapola 2024	Climate change and its impact on the mental health well-being of Indigenous women in Western cities, Canada	Western Canada	Perceived climate change: General worry	Qualitative: Narrative discussions
Cherry 2017	Effects of the Fort McMurray wildfires on the health of evacuated workers: follow-up of 2 cohorts	Fort McMurray, Alberta	Specific event: Wildfire	Questionnaire: HADS
Cunsolo 2020	“You can never replace the caribou”: Inuit experiences of ecological grief from caribou declines	Nunatsiavut and NunatuKavut, Newfoundland and Labrador	Perceived climate change: Rapidly changing climate environment (e.g. warming)	Qualitative: Interviews
Cunsolo Willox 2012	“From this place and of this place:” climate change, sense of place, and health in Nunatsiavut, Canada	Rigolet, Nunatsiavut, Newfoundland and Labrador	Perceived climate change: Changes in local weather precipitation, temperature, and weather patterns	Qualitative: Interviews Questionnaire: Higginbotham et al.’s (2007) Environmental Distress Survey
Cunsolo Willox 2013a	The land enriches the soul: on climatic and environmental change, affect, and emotional health and well-being in Rigolet, Nunatsiavut, Canada	Rigolet, Nunatsiavut, Newfoundland and Labrador	Perceived climate change: Changes in weather patterns, snow, ice, wildlife, vegetation, and fluctuations in seasonal temperatures	Qualitative: Interviews
Cunsolo Willox 2013b	Climate change and mental health: an exploratory case study from Rigolet, Nunatsiavut, Canada	Rigolet, Nunatsiavut, Newfoundland and Labrador	Perceived climate change: Changes in sea ice, snow, and seasonal temperature, as well as wildlife migration patterns due to warming temperatures	Qualitative: Interviews
Dodd 2018	Lived experience of a record wildfire season in the Northwest Territories, Canada	Yellowknife, N’Dilo, Detah and Kakisa, Northwest Territories	Specific event: Wildfires	Qualitative: Interviews
Fuentes 2020	Impacts of environmental changes on well-being in Indigenous communities in Eastern Canada	Quebec, Ontario (Ouje-Bougoumou, Opitciwan, Pikogan, Wahgoshig)	Perceived climate change: Slow onset changes due to climate change	Questionnaires: EDS, CD-RISC 10
Galway 2022	Exploring climate emotions in Canada’s Provincial North	Prince George, British Columbia, and Thunder Bay, Ontario	Perceived climate change: Increasing prevalence of climate-related extreme events (flooding and wildfires)	Questionnaire: Ad-hoc mental health survey
Galway 2023	Climate emotions and anxiety among young people (aged 16–25) in Canada: a national survey and call to action	Canada	Perceived climate change: General worry	Questionnaire: Ad-hoc climate emotions survey
Gignac 2003	Living with a chronic disabling illness and then some: data from the 1998 ice storm	Kingston, Ontario	Specific event: Ice storm	Questionnaire: CES-D
Harper 2015	Climate-sensitive health priorities in Nunatsiavut, Canada	Nunatsiavut, Newfoundland and Labrador	Perceived climate change: Changes in temperature, snow, ice, rain, or weather patterns over their lifetime	Qualitative: Interviews
Hayes 2020	Psychosocial adaptation to climate change in High River, Alberta: implications for policy and practice	High River, Alberta	Specific event: Flooding	Qualitative: Interviews
Hetherington 2018	Risk and protective factors for mental health and community cohesion after the 2013 Calgary flood	Calgary, Alberta	Specific event: Flood	Questionnaires: CES-D, SSAI, IES-R
Iskandar 1999	Crisis responses in a community exposed to a natural disaster: psychosocial impacts of the ice storm in Montreal	Montréal, Quebec	Specific event: Ice storm	Health records
Khalafzai 2021	Spring flooding and recurring evacuations of Kashechewan First Nation, northern Ontario, Canada	Albany river in Kashechewan First Nation, Ontario	Specific event: Flooding (numerous floods occurring in 1966, 1972, 1976, 1985 and 2006)	Qualitative: Interviews
Lavigne 2023	The effect modification of extreme temperatures on mental and behaviour disorders by environmental factors and individual-level characteristics in Canada	Alberta, Ontario (5 health regions in the province of Alberta and 35 health regions in the province of Ontario)	Specific event: Variations in outdoor ambient extreme temperatures (heat or cold exposures, varying from − 34.85 to 31.61 °C)	Health records: ICD-10
Lede 2021	The role of multiple stressors in adaptation to climate change in the Canadian Arctic	Paulatuk, Northwest Territories	Perceived climate change: Less predictable weather events, more severe and shorter onset (e.g. erosion, slumping, landslides, later sea ice freeze-up and earlier sea ice break-up, unexpected melting periods), changes in health/availability of wildlife	Qualitative: Interviews, observation
Lee 2023	Chronic diseases associated with mortality in British Columbia, Canada during the 2021 western North America extreme heat event	British Columbia	Specific event: Extreme heat	Health records: BC Vital Statistics Records (ICD-10 codes)
Léger-Goodes 2023	How children make sense of climate change: a descriptive qualitative study of eco-anxiety in parent-child dyads	Montréal and Sherbrooke, Quebec	Perceived climate change: General worry	Mixed methods: Qualitative interviews with children and parents, parent questionnaire
Lukacs 2023	The concerned steward effect: exploring the relationship between climate anxiety, psychological distress, and self-reported climate-related behavioural engagement	British Columbia	Perceived climate change: General worry	Questionnaires: Self-reported Climate-related Behavioural Engagement scores, Climate Change Anxiety Scale scores, and Kessler Psychological Distress scores
Lutz 2023	Eco-anxiety in daily life: relationships with well-being and pro-environmental behavior	Ottawa, Ontario	Perceived climate change: General worry	Qualitative: Daily diary study
Maltais 2000	Psychological and physical health of the July 1996 disaster victims: a comparative study between victims and non-victims	Saguenay, Quebec	Specific event: Flooding	Questionnaires: ABS, IES, BDI, GHQ-12
Maltais 2009a	Flood in Quebec: longitudinal study of long-term effects of psychological health	Saguenay, Quebec	Specific event: Flooding	Questionnaires: ABS, BDI, GHQ-12
Maltais 2009b	Consequences of the ice storm in Montérégie: psychosocial health and work performance of intervention team members	Montérégie, Quebec	Specific event: Ice storm	Qualitative: Interviews
Man 2024	Eco-depression and eco-anxiety among youth: a sex and gender analysis	Ontario	Perceived climate change: General worry	Questionnaires: 2021 Ontario Student Drug Use and Health Survey, Kessler Psychological Distress scale
Mao 2022	Posttraumatic stress disorder, major depressive disorder, and wildfires: a fifth-year postdisaster evaluation among residents of Fort McMurray	Fort McMurray, Alberta	Specific event: Wildfire	Questionnaires: PHQ-9, PCL-C
McDonald-Harker 2021	Social-ecological factors associated with higher levels of resilience in children and youth after disaster: the importance of caregiver and peer support	Fort McMurray, Alberta	Specific event: Wildfire	Mixed methods: Questionnaire (CYRM-28) and interviews
Middleton 2020	“We're people of the snow:” weather, climate change, and Inuit mental wellness	Nunatsiavut, Newfoundland and Labrador	Perceived climate change: Less predictable weather events, more severe and shorter onset, changes in health/availability of wildlife	Qualitative: Interviews
Middleton 2021	Temperature and place associations with Inuit mental health in the context of climate change	Nunatsiavut, Newfoundland and Labrador	Specific event: Exposure to elevated temperature	Health records: ICD-9 or ICD-10
Moosavi 2019	Mental health effects in primary care patients 18 months after a major wildfire in Fort McMurray: risk increased by social demographic issues, clinical antecedents, and degree of fire exposure	Fort McMurray, Alberta	Specific event: Wildfire	Questionnaires: PCL-5, PHQ-9, GAD-7, AUDIT, DUDIT
Norberg 2022	How do non-catastrophic natural disasters impact middle-aged-to-older persons? Using baseline Canadian Longitudinal Study on Aging data to explore psychological outcomes associated with the 2013 Calgary flood	Calgary, Alberta	Specific event: Flooding	Questionnaire & diagnosis: Primary Care Posttraumatic Stress Disorder (PC-PTSD) screening instrument; CES-D; anxiety diagnosed by a physician
Obuobi-Donkor 2022	Evaluating community resilience and associated factors one year after the catastrophic Fort McMurray flood	Fort McMurray, Alberta	Specific event: Flooding	Questionnaire: BRS
Obuobi-Donkor 2024a	2023 wildfires in Canada: living in wildfire regions in Alberta and Nova Scotia doubled the odds for residents to experience likely generalized anxiety disorder symptoms	Alberta and Nova Scotia	Specific event: Wildfire	Questionnaires: GAD-7, other ad-hoc questionnaires not described
Obuobi-Donkor 2024b	Mitigating psychological problems associated with the 2023 wildfires in Alberta and Nova Scotia: six-week outcomes from the Text4Hope program	Alberta and Nova Scotia	Specific event: Wildfire	Questionnaires: PHQ-9, GAD-7, PCL-C, BRS, WHO-5, PHQ-9
Oluwasina 2023	Evaluating the prevalence and correlates of major depressive disorder among residents of Fort McMurray, Canada, one year after a devastating flood	Fort McMurray, Alberta	Specific event: Flooding	Questionnaire: PHQ-9
Owusu 2022	Prevalence and determinants of generalized anxiety disorder symptoms in residents of Fort McMurray 12 months following the 2020 flooding	Fort McMurray, Alberta	Specific event: Flooding	Questionnaire: GAD-7
Pazderka 2021a	Model of posttraumatic growth in newly traumatized vs. retraumatized adolescents	Fort McMurray, Alberta	Specific event: Wildfire	Questionnaires: PHQ-A, HADS, CPSS, CRAFFT, CYRM-11
Pazderka 2021b	Collective trauma and mental health in adolescents: a retrospective cohort study of the effects of retraumatization	Fort McMurray, Alberta	Specific event: Wildfire	Questionnaires: PHQ-A, HADS, CPSS
Petrasek MacDonald 2015	Protective factors for mental health and well-being in a changing climate: perspectives from Inuit youth in Nunatsiavut, Labrador	Nunatsiavut, Newfoundland and Labrador	Perceived climate change: Changing sea ice and weather conditions	Qualitative: Interviews
Philippe 2020	Cognitive integration of personal or public events affects mental health: examining memory networks in a case of natural flooding disaster	Greater Montréal area, Quebec	Specific event: Flooding	Questionnaires: PHQ-9, BAI
Stone 2024	“It’s not something we like to think about because it’s so devastating”: understanding Eastern Canadian young women’s mental health in our changing climate	Nova Scotia	Perceived climate change: General	Qualitative: Interviews
Tilstra 2022	Exploring socio-environmental effects on community health in Edmonton, Canada to understand older adult and immigrant risk in a changing climate	Edmonton, Alberta	Specific event: Air pollution, temperature	Health records: ICD-10
Tupinier Martin 2024	The relationship between hot temperatures and hospital admissions for psychosis in adults diagnosed with schizophrenia: a case-crossover study in Quebec, Canada	Quebec	Specific event: Temperature	Health records: ICD-10
Turcotte-Tremblay 2024	Adolescents’ impairment due to climate anxiety is associated with self-efficacy and behavioural engagement: a cross-sectional analysis in Quebec (Canada)	Quebec	Perceived climate change: General worry	Questionnaires: Clayton et al.’s (2020) climate anxiety scale, GAD-7, National Longitudinal Study of Adolescent Health, School Connectedness Scale
Turmel 2023	Vagues de chaleur en contexte de changements climatiques – Annexe 2 Projet CASSSIOPÉE: capacité d’adaptation des systèmes de santé et services sociaux à protéger la santé mentale et le bien-être psychosocial des populations exposées à des événements météorologiques extrêmes	Quebec	Specific event: Extreme temperature (high)	Qualitative: Interviews
Valois 2020	Monitoring the evolution of individuals’ flood-related adaptive behaviours over time: two cross-sectional surveys conducted in the Province of Quebec, Canada	Quebec	Specific event: Flooding	Questionnaire: Ad-hoc mental health question
Vamvalis 2023	“We’re fighting for our lives”: centering affective, collective and systemic approaches to climate justice education as a youth mental health imperative	Ontario and British Columbia	Perceived climate change: General worry	Qualitative: Interviews and focus groups
Vergunst 2024	Association of youth climate change worry with present and past mental health symptoms: a longitudinal population-based study	Quebec	Perceived climate change: General worry	Questionnaires: Ad-hoc climate change worry measure, GAD-7, CES-D, Mental Health and Social Inadaptation Assessment for Adolescents
Verstraeten 2021	Maternal mental health after a wildfire: effects of social support in the Fort McMurray Wood Buffalo study	Fort McMurray, Alberta	Specific event: Wildfire	Questionnaires: IES-R, PDI, PDEQ, CD-RISC
Vida 2012	Relationship between ambient temperature and humidity and visits to mental health emergency departments in Quebec	Quebec (16 health administrative regions of Quebec except northern Quebec)	Specific event: Extreme temperatures (high and low)	Health records: ER visits labelled mental and psychosocial problems
Villeneuve 2023	Daily changes in ambient air pollution concentrations and temperature and suicide mortality in Canada: findings from a national time-stratified case-crossover study	Canada	Specific events: Air pollution, temperature	Health records: Canadian Vital Statistics Death Database ICD-10 codes for intentional self-harm underlying death
Wang 2014	Acute impacts of extreme temperature exposure on emergency room admissions related to mental and behaviour disorders in Toronto, Canada	Toronto, Ontario	Specific event: Extreme temperatures (high and low)	Health records: ICD-10 codes related to mental and behaviour disorders, schizophrenia, neurotic disorders, and mental and behaviour disorders due to psychoactive substance use
Woodhall-Melnik 2019	Perceptions of mental health and wellbeing following residential displacement and damage from the 2018 St. John River flood	Southern New Brunswick	Specific event: Flooding	Qualitative: Key informant interviews and focus groups
Yoon 2024	Responding to the heat and planning for the future: an interview-based inquiry of people with schizophrenia who experienced the 2021 heat dome in Canada	British Columbia	Specific event: Extreme heat	Qualitative: Interviews

**Abbreviations:** ABS, Affect Balance Scale; AUDIT, Alcohol Use Disorder Identification Test; BAI, Beck Anxiety Inventory; BC, British Columbia; BDI, Beck Depression Inventory; BRS, brief resilience scale; CAGE, Cut down, Annoyed, Guilty, and Eye-opener; CAPS, Clinician-Administered PTSD Scale; CCHS, Canadian Community Health Survey; CD-RISC, Connor–Davidson Resilience Scale; CES-D, Center for Epidemiologic Studies - Depression Scale; CPSS, Child PTSD Symptom Scale; CRAFFT, CRAFFT Questionnaire; CYRM, Child and Youth Resilience Measure; DSM-5, Diagnostic and Statistical Manual of Mental Disorders, 5th Edition; DUDIT, Drug Use Disorder Identification Test; EDS, Environmental Distress Scale; GAD, Patient Health Questionnaire Generalized Anxiety Disorder Scale; GHQ, General Health Questionnaire; HADS, Hospital Anxiety and Depression Scale; ICD, International Statistical Classification of Diseases and Related Health Problems; IES-R, Impact of Event Scale–Revised; ISI, Insomnia Severity Index; MINI, Mini International Neuropsychiatric Interview; PCL-5, PTSD Checklist for DSM-5; PCL-C, PTSD Checklist for DSM-5, civilian version; PDEQ, Peritraumatic Dissociative Experiences Questionnaire; PDI, Peritraumatic Distress Inventory; PHQ-9, Patient Health Questionnaire; PHQ-A, Patient Health Questionnaire, Adolescent version; PSQI, Pittsburgh Sleep Quality Index; PSQI-A, Pittsburgh Sleep Quality Index–Addendum; PTCI, Posttraumatic Cognitions Inventory; PTSD, posttraumatic stress disorder; SRQ, Self-reporting Questionnaire; SSAI, Spielberger State Anxiety Inventory; WCQ, Ways of Coping Questionnaire; WHO-5, World Health Organization-5 Well-being Index.

**Table 2:** Main findings from included articles in climate change and mental health research in Canada review

**Author**	**Length of impact**	**Population**	**Main findings**
Adu 2022	Long term	186 residents of Fort McMurray in 2021 (5 years post-wildfire). 86% female, 82% > 40 years, 94% employed, 71% in a relationship, 44% no significant loss from fire.	Younger age and having PTSD showed significant association with low resilience. Anxiety and depressive disorders are not correlated with resilience.
Adu 2024	Intermediate term	298 complete survey respondents aged 18 years and above, from Alberta and Nova Scotia.	Unemployment and a history of mental health diagnosis predicted likely PTSD, while unemployment was associated with low resilience during the wildfire.
B. Agyapong 2022a	Long term	186 residents of Fort McMurray in 2021 (5 years post-wildfire). 86% female, 53% > 40 years, 71% in a relationship, 90% lived in Fort McMurray during the wildfire.	Increased prevalence of PTSD but not major depressive disorder or generalized anxiety disorder in employees of other organizations compared to school board staff. Employer type did not significantly predict PTSD. No significant differences between school employees vs. others with respect to those who received counselling, those willing to receive counselling, those taking antidepressants. No difference in resilience levels between employee groups.
B. Agyapong 2022b	Long term	186 residents of Fort McMurray in 2021 (5 years post-wildfire).	Individuals who experienced Fort McMurray wildfires and/or flooding in addition to COVID-19 have an increased likelihood of generalized anxiety disorder, major depressive disorder, PTSD and low resilience.
B. Agyapong 2022c	Long term	186 residents of Fort McMurray in 2021 (5 years post-wildfire). 86% female, 50% were < 40 years, 95% employed, 72% in a relationship.	33% of respondents use antidepressants, 11% use sleeping medication. 40% of respondents received mental health counselling in the past year. 27% of respondents abused alcohol, and 11% abused marijuana. 46% had depression, 44% had anxiety, 41% had likely PTSD, 40% low resilience, and 18% self-harm thoughts among female respondents. Significant predictors of suicidal ideation and self-harm thoughts: desire to receive mental health counselling, and alcohol abuse.
V. Agyapong 2018	Intermediate term	484 residents of Fort McMurray during the wildfire. 75% > 26 years, 67% female, 87% employed or in school, 64% in a relationship, 48% no significant loss from the fire.	Approximately 20% had generalized anxiety symptoms, and significant predictors included: being young female, staying in rentals, relocated to a different home, being exposed to media coverage relating to the wildfire, having pre-existing mental health conditions, and receiving inadequate support.
V. Agyapong 2020	Long term	197 staff at two school districts in Fort McMurray 1.5 years after the wildfire. 85% female, 47% > 40 years, 86% in a relationship, 42% teacher, 19% home destroyed by fire.	Likely major depressive disorder rates in school staff is 18%. 16% of school staff had a likely generalized anxiety disorder diagnosis 18 months after the fire. 10% of staff had PTSD after the fire. Likely major depressive disorder, generalized anxiety disorder and PTSD showed significant association with problematic drug use but not with high-risk alcohol drinking.
V. Agyapong 2021	Intermediate term	486 residents of Fort McMurray during the wildfire. 66.3% female, 75% > 26 years, 87% employed or in school, 64% in a relationship, 48% no significant loss from the fire.	1-month prevalence of likely PTSD 6 months post-disaster is 12.8% (1.57 OR higher for females vs. males). Significant differences from those who suffered PTSD: living in a different home, history of anxiety disorder, receiving inadequate support, receiving counselling. Not significant predictors: complete home loss, loss of property in general, being younger, being female. Respondents with likely PTSD more likely to self-report increased drug use but not alcohol use.
Auger 2000	Intermediate term	Low family annual income < $25 000 (~60%), mostly female population.	4 months post-flood, nearly 20% of victims met the diagnostic criteria of PTSD compared to nearly 4% of controls.
Beaumier 2010	Long term	Semi-structured interviews with 36 Inuit women, 5 focus groups with 19 Inuit women and interviews with 13 key informants. 72% of women were unemployed. 48% of women were full- or part-time hunters.	Changing climatic conditions are exacerbating the food security implications of a decline in the number of full-time hunters and declining number of youths hunting full-time. Adapting to changing accessibility and safety of hunting requires well-developed understanding of ice conditions, weather patterns and animal movements, which only full-time experienced hunters have. Consequently, climate change is reinforcing the trend of reduced participation in hunting among youth with implications for country food availability. With reduced availability of country foods, sharing networks are negatively impacted and it is often women who suffer the most by reducing food consumption, particularly in households without the financial ability to afford store foods.
Belanger 2014	Short and long term	3485 respondents living in very disadvantaged dissemination areas of the nine most populated cities within Quebec.	Prevalence of self-reported adverse mental health impacts during very hot and humid summer conditions was 17.8% among participants.
Belleville 2019	Short term	379 respondents living in Fort McMurray completed questionnaires between July and September 2016. 77% female, mean age was 40.10 years old (SD = 12.2), 72% in a relationship, 61% of respondents had children. 56 respondents completed the interview (51% females). Mean age was 43.1 years (SD = 14.7), 49% in a relationship, 62% had children.	60% of respondents reported significant PTSD symptoms. 33% of participants have major depression disorder, 27% have somatoform disorder, 27% have other anxiety disorder, 17% have alcohol abuse.
Belleville 2021	Intermediate and long term	Current or former residents of Fort McMurray who had been evacuated from the 2016 fires. Data collected during May and July 2017. Mean age 44.1 years (SD = 12.7), 55.5% female, 73.9% White, 73.2% in a relationship, 76.8% employed.	Insomnia was the most common probable diagnosis 1 year after the fire (28.5%). PTSD (15.4%), major depressive disorder (15.0%), generalized anxiety disorder (14.2%) and substance use disorder (7.9%) were also reported 1-year post-fire. Approximately 37.7% had at least one probable diagnosis. Having two pre-existing conditions was a significant predictor to experiencing all five types of psychopathologies. Reduced work, reduced social life, poorer current health status, increase in drug and alcohol use, and level of stress were associated with more severe symptoms.
Brown 2019a	Long term	3070 children and youth in grades 7–12 attending school in Fort McMurray. 48% female, mean age 14.3 years (SD = 1.8).	The findings indicate rates of probable PTSD (37%), probable depression (31%), probable anxiety (27%), and probable alcohol/substance use disorder (15%). The findings indicate that students directly impacted by the fire had significantly higher scores on scales measuring symptoms related to PTSD, depression, anxiety and substance misuse. These students also had significantly lower scores for self-esteem, quality of life and resilience.
Brown 2019b	Long term	3070 children and youth in grades 7–12 attending school in Fort McMurray. 48% female, mean age 14.3 years (SD = 1.8). Comparison group: 2796 children and youth in Red Deer; 48% female, mean age 14.8 years (SD = 1.7).	Depression scores were significantly higher in Fort McMurray, as were rates of probable depression, suicidal thinking and tobacco use. Self-esteem scores and quality of life scores were significantly lower in Fort McMurray. Anxiety scores were significantly higher in Fort McMurray. Alcohol/substance misuse scores were not significantly different.
Brown 2021	Long term	Survey conducted in November 2017, 2018, 2019. 9376 students from grades 7–12 in Fort McMurray. 47.3% female, 48.6% male, 1.7% other, 2.4% preferred not to say. Mean age of participants was 14.3 years (SD = 1.8 years).	The results indicate a slow but statistically significant trend of worsening mental health from 2017 to 2019, including increased symptom scores and increased rates of probable PTSD, depression, anxiety, drug use and alcohol use. Quality of life and self-esteem scores also decreased from 2017 to 2019. Tobacco use and resilience scores did not change significantly.
Bunce 2016	Long term	42 Inuit women living in Iqaluit in June 2015 (median age = 39 years; 61% employed).	Three key traditional activities were identified as being exposed and sensitive to changing conditions: berry picking, sewing and the amount of time spent on the land. Several coping mechanisms were described to help women manage these exposure sensitivities, such as altering the timing and location of berry picking and importing seal skins for sewing. The adaptive capacity to employ these mechanisms differed among participants; however, mental health, physical health, traditional/western education, access to country food and store-bought foods, access to financial resources, social networks and connection to Inuit identity emerged as key components of Inuit women’s adaptive capacity.
Chapola 2024	Short and long term	15 Indigenous women across Western Canadian cities.	Climate disaster–induced impacts and uneven exposure to various disastrous events are affecting conscious and subconscious thinking, creating fear and anxiety among Indigenous women. Moreover, reflecting on the adverse mental health impacts and changes to their sense of place (belongingness), many Indigenous women (co-researchers) highlighted the additional layer of mental stress, mood swings, emotional disorders, etc., experienced by Indigenous women and community members who are already facing significant changes due to the loss of land-based activities, their ancestral territories and their knowledge systems. Many co-researchers argue that this has been further aggravated due to the lack of a proper understanding of the complexity of Indigenous health issues and institutionalized mental health support networks in the territory.
Cherry 2017	Intermediate term	130 participants (62% male, 31.5% above the age of 46 years, 42% married). 59% have home established in Fort McMurray.	Thirty-seven (33.9%) of the participants who were in Fort McMurray on May 3 reported a health condition, including respiratory symptoms (n = 17) and mental ill health (n = 17), immediately after the fire. At follow-up, a mean of 102 days after the fire, 11 participants (10.1%) reported a fire-related health condition, including mental ill health (n = 8). There was no difference before and after the fire in use of alcohol, cigarettes, recreational drugs or medication. Of the 90 participants evacuated, 15 (16.7%) had scores indicative of moderate or severe anxiety or depression. Those evacuated had significantly higher mean anxiety (*p* = 0.01) and depression (*p* = 0.04) scores than those not evacuated. Regression modelling showed that anxiety scores were higher for women, with longer time since the fire and with evacuation to a motel. Depression scores were higher for women and with financial loss because of lack of work.
Cunsolo 2020	Long term	105 interviews with Inuit participants.	The caribou play a central role for culture and cultural continuity, livelihoods and sustenance, long-standing intergenerational knowledge sharing, spiritual practices, social cohesion, physical health, and mental and emotional wellness. While Inuit in both the Nunatsiavut and NunatuKavut regions of Labrador have been actively researching, monitoring and making management recommendations about caribou, the rapid decline in caribou, the current ban on harvesting and the resulting loss of access to caribou have led to experiences of strong emotional responses, such as the experiences of sadness, anger, frustration, shock, depression and grief.
Cunsolo Willox 2012	Long term	72 interviews with Inuit participants (60% female) including children, adolescents and adults.	The ﬁndings illustrated that climate change is negatively affecting feelings of place attachment by disrupting hunting, ﬁshing, foraging, trapping and travelling, and changing local landscapes—changes which subsequently impact physical, mental and emotional health and well-being. These results also highlight the need to develop context-speciﬁc climate-health planning and adaptation programs and call for an understanding of place attachment as a vital indicator of health and well-being and for climate change to be framed as an important determinant of health.
Cunsolo Willox 2013a	Long term	57 Inuit participants (42% male).	From data gathered through a multiyear, community-driven project in Rigolet, Nunatsiavut, Labrador, Canada; however, it is evident that the emotional consequences of climate change are extremely important to Northern residents. Participants shared that these changes in land, snow, ice and weather elicit feelings of anxiety, sadness, depression, fear and anger, and impact culture, a sense of self-worth and health. This article analyzes the affective dimensions of climatic change and argues that changes in the land and climate directly influence emotional health and well-being. Narratives of Inuit lived experiences will be shared through data from interviews, the concept of ecological affect will be introduced and implications for climate-health research and programming will be discussed.
Cunsolo Willox 2013b	Long term	67 members from the Inuit population (60% females) between January 2010 and October 2010.	Participants reported that changes in weather, snow and ice stability and extent, and wildlife and vegetation patterns attributed to climate change were negatively impacting mental health and well-being due to disruptions in land-based activities and a loss of place-based solace and cultural identity. Participants reported that changes in climate and environment increased family stress, enhanced the possibility of increased drug and alcohol usage, amplified previous traumas and mental health stressors and were implicated in increased potential for suicide ideation. These exploratory findings indicate that climate change is becoming an additional mental health stressor for resource-dependent communities and provide a baseline for further research.
Dodd 2018	Short and long term	30 interviews with Indigenous community in Northwest Territories.	Interviewees reported how their experiences of evacuation and isolation as well as feelings of fear, stress and uncertainty contributed to acute and long-term negative impacts for their mental and emotional well-being. Prolonged smoke events were linked to extended time indoors and respiratory problems. Livelihood and land-based activities were disrupted for some interviewees, which had negative consequences for mental, emotional and physical well-being. Individual and community stories of adaptation and resilience prior to and during the summer, including the opening of indoor recreational spaces, were shared; however, there was consensus about the need for improved risk communication and coordination at the community and territorial levels to address similar events in the future.
Fuentes 2020	Long term	251 participants (69% older than 35 years old). 29% Ouje-Bougoumou, 15% Wahgoshig, 26% Pikogan, 29% Opitciwan in Eastern Canada.	The felt impacts of environmental changes increased with age but were lower for participants with higher quality of life. The effect of resilience was opposite to expectations: more resilient participants felt more impacts. This could be because less resilient individuals ceased to go on the land when environmental changes exceeded a given threshold; thus, only the most resilient participants could testify to the impacts of acute changes.
Galway 2022	Long term	627 participants (mean age = 57 years; 41% male; 90% White) in Thunder Bay, Ontario and Prince George, British Columbia.	Results show high levels of emotional response to climate change overall, with worry and frustration as those emotions reported by the highest percentage of participants. There is also a significant difference in climate emotions between men and women. A notable limitation of the data is their underrepresentation of Indigenous peoples.
Galway 2023	Short and long term	1000 young people (aged 16–25) across Canada.	78% reported that climate change impacts their overall mental health and 37% reported that their feelings about climate change negatively impact daily functioning. Young people most commonly report feeling afraid (66%), sad (65%), anxious (63%), helpless (58%) and powerless (56%). The least commonly reported feelings were grief (34%), optimism (21%) and indifference (20%).
Gignac 2003	Intermediate and long term	59 older adults with osteoarthritis or osteoporosis in Kingston who experienced the ice storm and 55 controls (older adults).	Older adults who reported greater helplessness and lost independence prior to the storm reported significantly greater ice storm stress and rumination and were more likely to report that the storm affected their condition. In addition, participants exposed to the ice storm reported significant changes in disability and pain nearly a year and a half later, compared to matched controls. These results suggest that older adults with chronic physical illness may be particularly vulnerable when faced with additional stressful events.
Harper 2015	Long term	11 interviews with regional health representatives in Nunatsiavut (82% adults, 82% female), 11 workshops with Rigolet community members (64% adults, 45% female) and 187 surveys with Rigolet members (57%–62% adult, 41%–54% female).	Climate-sensitive health factors were described in terms of inter-relationships between environmental and social determinants of Inuit health. The climate-sensitive health priorities for the region included food security, water security, mental health and well-being, new hazards and safety concerns and health services and delivery.
Hayes 2020	Long term	14 interviews with local leaders, focus groups with 14 front-line workers, and 18 interviews with community members who self-identified as marginalized (low-income, female, youth, elderly pre-existing health concerns, racialized) who were in High River during the 2013 flood.	Findings of this study suggest (1) the long-term psychosocial impacts of extreme weather and climate change require sustained recovery interventions rooted in local knowledge and interdisciplinary action; (2) there are unintended consequences related to psychosocial interventions that can incite complex emotions and impact psychosocial recovery; and (3) perceptions of mental health care, among people exposed to climate-related trauma, can guide climate change and mental health response and recovery interventions.
Hetherington 2018	Intermediate term	923 women. Mean age = 34 years; approximately 80% had at least post-secondary education; had a higher income (> $80K); were Caucasian and born in Canada. About 27% had previously been exposed to a natural disaster.	Most respondents in the sample did not experience major psychological distress 5 months after the flood. Elevated anxiety before the flood was associated with 2.49 increased odds of experiencing high levels of posttraumatic stress, regardless of whether respondents lived in a flood-risk community or not. A higher level of exposure to the flood (living in a community at risk of flooding or experiencing loss or damage to property or services) was not the only factor that was associated with high levels of posttraumatic stress or other mental health outcomes after the flood. Women with underlying mental health conditions may be more vulnerable to the psychological impacts of a natural disaster regardless of their level of exposure.
Iskandar 1999	Short term	Extracted medical records from psychiatric patients pre (123), during (January 6–26, 1998: 165), post (145) disaster and 51 medical patients.	165 patients were kept under observation in the emergency department with an average stay of 105.7 hours. In the group of 51 patients hospitalized at Douglas Hospital, 40% were characterized by the combination of mood disorders, somatic problems and lack of social support. About 16% of this group was characterized by the following combination: psychotic disorders, somatic problems and lack of social support. Only 8% of patients with mood disorders and 8% with psychosis were hospitalized; these types of patients were well supported socially and did not suffer from any somatic problems.
Khalafzai 2021	Long term	Interviews with 41 participants from Kashechewan First Nation in Northern Ontario (68% male, 90% Band member).	Results show that spring flood risk has significantly increased the community’s physical and socio-cognitive vulnerability. Flooding frequently impacts community infrastructure, traditional spring hunting and harvesting and the local economy. It also significantly increases the stress and anxiety of residents every year as spring approaches. Dealing with the regular flooding risk and recurring emergency experiences have improved the community’s disaster preparedness and coping capacity, but residents’ evacuation experiences negatively affect their well-being during and after the evacuations. Short-term actions addressing the physical vulnerability of individuals living with heightened hazard risks often enhance their ability to build resilience and cope effectively in the immediate future.
Lavigne 2023	Short term	Extracted ED admissions from across Ontario and Alberta. 60.9% of ED visits from adults over the age of 30 years, 53.2% female, 18.3% materially and socially deprived.	Cumulative exposure to extreme heat over 0–5 days (odds ratio [OR] = 1.145) was associated with emergency department (ED) visits for any mental and behavioural disorder (MBD). However, cumulative exposure to extreme cold was associated with lower risk of ED visits for any MBD (OR = 0.981). Heat was also found to be associated with ED visits for specific MBDs such as substance use disorders, dementia, neurotic disorders, schizophrenia and personality behaviour disorder. Individuals with pre-existing mental health conditions, those exposed to higher daily concentrations of NO_2_ and O_3_ and those residing in neighbourhoods with greater material and social deprivation were at higher risk of heat-related MBD ED visits. Increasing tree canopy coverage appeared to mitigate risks of the effect of heat on MBD ED visits.
Lede 2021	Long term	28 interviews with individuals from Paulatuk, Northwest Territories, Canada (53.6% male, 42.9% over the age of 50 years).	In the context of subsistence harvesting, climatic stressors have affected access to, and the availability of, some fish and wildlife and are making travel conditions more unpredictable and dangerous. These stressors are being experienced at the same time as societal stressors such as financial and social barriers to participating in subsistence, challenges with local schooling, lifestyle changes, housing shortage and overcrowding, and addiction. Many of the coping strategies used by people in Paulatuk to deal with stressors involve trade-offs, such as leaving the community for school or leaving school to participate in subsistence and switching species harvested in response to a decline in one species, which has undermined resilience to other stressors.
Lee 2023	Short term	Comparison of 1614 deaths from 25 June to 2 July 2021 with 6524 deaths on the same dates from 2012 to 2020 to examine differences in the prevalence of 26 chronic diseases between the two groups.	There was a pronounced increase in odds of death for those with schizophrenia. This increase was observed among all deaths and the three extreme heat event subgroups: heat-related deaths, deaths with pending cause and non-heat-related deaths. Depression was associated with increased odds of extreme heat event mortality among heat-related deaths, and substance use disorder was associated with increased odds among deaths with pending cause.
Léger-Goodes 2023	Short and long term	12 families, with 12 parents (n = 9 women, n = 2 men, n = 1 non-binary) and 15 children (n = 9 girls, n = 6 boys) between the ages of 8 and 12 years.	While children appear to experience emotions such as sadness, anger and apprehension that constitute eco-anxiety, they also reported strategies to manage these difficult emotions, especially when their parents are aware of their concerns. Children expressed feelings of stress related to climate change and of being overwhelmed by their emotions and fear of their future. The results also suggest that children’s expression of eco-anxiety resembles that of adults.
Lukacs 2023	Short term	1553 participants (33.6% above 45 years; 49.6% men, 79% White, 73.8% in a relationship).	Higher Climate Change Anxiety Scale scores and higher Behavioural Engagement scores were both associated with greater psychological distress. Findings suggest that self-reported behavioural engagement and climate anxiety are correlated, a phenomenon referred to as the concerned steward effect. However, the association becomes attenuated among those with high levels of distress, perhaps driven by a diminishing return of behavioural engagement or difficulties of behavioural engagement among those with high distress.
Lutz 2023	Short term	132 Carleton University undergraduate students (72.6% female; 41.9% White; mean age = 21.2 years, SD = 4.97 years).	Trait reports and two weeks of daily reports (n = 1439) on eco-anxiety, positive and negative affect, meaning in life, and pro-environmental behaviour revealed that at the trait level, average scores on eco-anxiety were low; yet, higher scores were associated with less positive affect and more negative affect and pro-environmental behaviour. Average scores on eco-anxiety were even lower at the state level but nonetheless exhibited notable within-person variability. On days that people felt greater eco-anxiety, they also reported greater negative affect and pro-environmental behaviour. Lagged analyses from one day to the next provided some evidence that eco-anxiety increases future negative affect. No significant relationship between eco-anxiety and meaning in life emerged at both levels of analysis.
Maltais 2000	Long term	Residents of Saguenay–Lac-St-Jean of 177 flood victims/168 non-flood victims from July 1996. Mean age: 18+ years at the time of the flood, 50%–60% high school diploma and 40%–50% had post-secondary education.	After a natural disaster involving significant property damage, the psychological health of the victims is significantly worse than that of the non-disaster victims. In this case, regardless of gender, the differences are significant. There was a significant increase in the consumption of prescribed and non-prescribed medications. More than two years after the disaster, more victims than non-victims present somatic symptoms, anxiety, depression, PTSD and lower levels of psychological well-being.
Maltais 2009a	Long term	Residents of Saguenay–Lac-St-Jean followed up eight years after the July 1996 flood (130 flood victims; 88 non-flood victims). Similar demographic trends as Maltais (2000).	In urban areas, the results show a decrease in posttraumatic manifestations and depression among victims. However, significant differences between victims and non-victims exist regarding posttraumatic stress manifestations and depression, social dysfunction, somatic symptoms, anxiety and insomnia. Over the years, victims had improved scores on the GHQ-28 and the Affect balance scale while maintaining a significant difference from non-victims on the GHQ-28. In rural areas, the scores of the GHQ-28 and on scales measuring the presence of somatic symptoms, anxiety and insomnia and severe depression have improved. No significant difference was noted for psychological well-being, the presence of depressive behaviour, posttraumatic stress and social dysfunction.
Maltais 2009b	Long term	First responders and support personnel surveyed in autumn 1999 regarding their experiences during and after the ice storm crisis in Montérégie, Quebec. A total of 57 (30 professionals, 27 volunteers).	One year after the ice storm, first responders and support personnel were more affected in the aftermath of the crisis than during the storm. The persistence of physical and psychological fatigue caused a loss of energy and interest in their professional and personal activities. They do not seem to be affected by psychological trauma which could lead to incapability to work.
Man 2024	Short term	Sex-based analysis: n = 1029; gender-based analysis: n = 623 of Ontario-based youth using the 2021 Ontario Student Drug Use and Mental Health Survey.	Gender-diverse youth and youth assigned female at birth may be especially likely to experience eco-depression and eco-anxiety.
Mao 2022	Long term	186 participants from Fort McMurray between 2014 and 2021 (85.5% females; 52.7% > 40 years of age; 94.1% employed; 71% in a relationship).	The overall prevalence of MDD symptoms in the study sample was 45.0%. Four variables independently predicted MDD symptoms in the multivariate logistic regression model, including: unemployed (OR = 12.39), have received a mental diagnosis of MDD (OR = 4.50), taking sedative-hypnotics (OR = 5.27) and willingness to receive mental health counselling (OR = 4.90). The prevalence of likely PTSD among the respondents was 39.6%.
McDonald-Harker 2021	Long term	100 children and youth between 5 and 18 years old who experienced the 2016 Fort McMurray wildfire. The participants had a mean age of 11 years and 55% were female.	Quantitative findings reveal higher than average levels of resilience among the participants compared to normative scores. Qualitative findings suggest high levels of resilience were associated with both caregiver factors (specifically physical caregiving) and individual factors (primarily peer support).
Middleton 2020	Long term	Gathered from 2012–2013, 116 participants interviewed across all five communities in Nunatsiavut (96 community members, 20 health professionals).	Results indicated that weather impacted mental wellness through three key factors: 1) shaping daily lived experiences including connection to place and other determinants of well-being; 2) altering mood and emotion on a transient basis; and 3) seasonally influencing individual and community health and well-being.
Middleton 2021	Short term	All mental health–related clinic visits from five Nunatsiavut community clinics from 1 January 2012 to 31 December 2018.	Regionally, the incidence rate of mental health–related community clinic visits was greater after two weeks of warm average (i.e. above −5°C) temperatures compared to temperatures below −5°C (Incidence Rate Ratio [IRR] −5 ≤ 5°C = 1.47; IRR 6 ≤ 15°C = 2.24; IRR > 15°C = 1.73) and the incidence rate of mental health–related clinic visits was lower when the number of consecutive days within −5 to 5°C ranges (i.e. temperatures considered to be critical to land use) increased (IRR = 0.96), adjusting for seasonal and community effects. Community-specific models, however, revealed that no two communities had the same association between meteorological conditions and the incidence rate of daily mental health–related visits.
Moosavi 2019	Long term	290 patients in Fort McMurray 18 months after the wildfire (79.6% employed, 44.8% over the age of 40 years, 45.1% male, 72.1% in a relationship).	The 1-month prevalence rate for “likely PTSD” was 13.6%, “likely MDD” was 24.8% and “likely GAD” was 18.0%. Compared to self-reported prevalence rates before the wildfire (0%, 15.2% and 14.5% respectively), these were increased for all diagnoses. PTSD: History of anxiety disorder and received counselling had odds ratios (ORs) of 5.80 and 7.14, respectively. MDD: Age, witnessed the burning of homes, history of depressive disorder and receiving low-level support from friends and family had ORs of 2.08, 2.29, 4.63 and 2.5, respectively. GAD: Fearful for their lives or the lives of friends/family, history of depressive disorder and history of anxiety disorder had ORs of 3.52, 3.04 and 2.68, respectively. There were also associations between individuals with a likely psychiatric diagnosis and those who also had likely alcohol or drug abuse/dependence.
Norberg 2022	Intermediate term	826 individuals who completed the CLSA (59.3% aged 45–64 years, 61.5% female, 65.3% in a relationship, 93.3% White) in Calgary during the flood.	They compared baseline CLSA data collected on Calgary participants during the 6 months prior to and following the flood. There were no statistically significant differences in the relative proportions of CLSA participants pre- and post-flood experiencing three or more PTSD symptoms (5.0% pre-flood vs. 4.6% post-flood), fair or poor self-rated mental health (5.5% vs. 5.8%), fair or poor self-rated general health (11.2% vs. 8.9%), dissatisfaction with sleep (27.9% vs. 30.2%), lower level of life satisfaction (10.6% vs. 14.9%), significant depressive symptoms (14.4% vs. 15.6%), diagnosed anxiety disorder (5.7% vs. 5.2%) and lower levels of functional support (45.9% vs. 49.9%). After adjusting for socio-demographic covariates (age, sex, total household income, living alone), individuals living in evacuated areas pre-flood were significantly less likely to report a diagnosis of an anxiety disorder (OR = 0.28, *p* = 0.03) (Table 3). Post-flood, this statistically significant difference disappeared (OR = 0.83, *p* = 0.72). This was explained by an increase in the prevalence of anxiety disorders among respondents in evacuated communities (from 2.4% pre-flood to 4.7% post-flood) and a decline among those in non-evacuated communities (from 8.1% pre-flood to 5.5% post-flood).
Obuobi-Donkor 2022	Long term	Data collected in 2021 among residents in Fort McMurray (1 year after the flood). 186 respondents (93% over the age of 26 years, 67% females, 91% employed and 71% in a relationship).	The prevalence of low resilience was 37.4%. Respondents under 25 years were nearly 26 times more likely to show low resilience (OR = 0.04) than respondents 40 years and above. Respondents with a history of depression (OR = 0.25) and a history of anxiety (OR = 0.21) were nearly four to five times more likely to show low resilience than those without a history. Participants residing in the same house before the flood were almost 11 times more likely to show low resilience (OR = 0.1) than those who relocated.
Obuobi-Donkor 2024a	Short term	298 participants (85% female; mean age = 48.4 years, SD = 14; 28.3% under 40 years of age; 83.5% White; 56.4% in a relationship; 63.5% employed).	The research has underscored a significant association between exposure to wildfire-affected areas and heightened anxiety levels among the affected population. The study revealed that the prevalence rate of moderate to severe anxiety during the wildfire was 41.9%.
Obuobi-Donkor 2024b	Short term	306 subscribers to the Text4Hope program (130 subscribers partially or fully completed both the baseline and six-week surveys). 84% White, 53.9% in a relationship and 63% employed.	Results from the longitudinal study show that the mean scores on the WHO-5 Well-being Index, PHQ-9, GAD-7 and PCL-C and the ninth question of the PHQ-9, which measures the intensity of suicidal ideation, were statistically significantly improved at six weeks compared to their corresponding baseline scores, except on the BRS.
Oluwasina 2023	Long term	186 respondents in Fort McMurray one year after the flood (85.5% female, 52.7% were above 40 years of age).	The prevalence of mild to severe depression among the respondents was 53.7%. Respondents who reported that they were unemployed were 12 times more likely to have moderate to severe depression (OR = 12.16).
Owusu 2022	Long term	186 respondents in Fort McMurray one year after the flood (85.5% female, 81.6% above 40 years of age, 71% in a relationship, and 94.1% employed).	The prevalence of likely GAD was 42.5%. Predictors of likely GAD among respondents included positive employment status (OR = 30.70).
Pazderka 2021a	Long term	Previously traumatized (n = 295) and wildfire trauma alone (n = 740) adolescents in Fort McMurray after the 2016 wildfire.	Together, relationships between Age and Self Efficacy, and Self Efficacy and Hopelessness suggest that, for individuals experiencing trauma for the first time, there exists an age-related boost in confidence in their ability to handle things, which was negatively related to Hopelessness. For the wildfire group, the oldest adolescents showed higher mean levels of confidence; for the prior trauma group, these age-related improvements did not exist.
Pazderka 2021b	Long term	Data collection among children and adolescents in Fort McMurray in 2019 (n = 295 previous trauma group, n = 740 wildfire trauma alone group).	Individuals who experienced prior trauma had significantly higher rates of both depression and PTSD, but not in anxiety or risk of suicide.
Petrasek MacDonald 2015	Long term	Interviews conducted with youth aged 15–25 from the five communities in the Nunatsiavut region.	Five key protective factors were identified as enhancing their mental health and well-being: being on the land; connecting to Inuit culture; strong communities; relationships with family and friends; and staying busy. Changing sea ice and weather conditions were widely reported to be compromising these protective factors by reducing access to the land and increasing the danger of land-based activities.
Philippe 2020	Short term	224 participants from the general population (mean age = 36.6 years, 74% female) directly or indirectly affected by a natural flooding disaster.	Results showed that flood-affected individuals reported poorer mental health compared to the unaffected. However, both affected and unaffected individuals who had encoded a current floods-related event in memory as need-satisfying or who had embedded such an event in need-satisfying memory networks showed better mental health over time. These results stayed the same after controlling for the effect of various demographics and dispositional emotion regulation styles.
Stone 2024	Long term	9 young women between the ages of 16 and 28 years of age.	First, participants embraced the discourse of hopelessness and pessimism surrounding climate change and our future, with the issue seeming too uncertain, grave and anxiety-inducing to think about. Second, participants believed that subject positions, such as gender, socioeconomic status and race, impact how people experience climate change.
Tilstra 2022	Short term	Adults in Edmonton that had health events between 1 January 2015 and 31 December 2018.	Measures of heat exposure and increased temperature had positive associations with mental health, but the results were inconsistent. Rain was the only climate-related variable that was consistently significant across community-level vulnerability models, with a 5.202 mm interquartile range (IQR) increase in rain associated with approximately a 21% decrease in the rate of mental health events. Increasing industrial emissions was only a significant risk factor for higher percentages of immigrants. When the fraction of total immigrants was 5%, a 3.8 t/km^2^ (IQR) increase in industrial emissions was associated with a prevalence rate ratio (PRR) of 0.972 for mental health events compared to a PRR of 1.186 when the fraction of total immigrants increased to 45%. The protective effect of a higher proportion of immigrants diminished at higher levels of industrial emissions. Like other outcomes, increasing concentrations of O_3_ demonstrated protective effects in main effects models.
Tupinier Martin 2024	Short term	30 649 adults diagnosed with schizophrenia (59.7% men; 20.8% over 50 years of age).	Overall, analysis of the cumulative relationship between mean temperatures and hospital admissions (HAs) for psychosis over six days pointed towards an increase in HAs, although the results were non-statistically significant. There was a statistically significant increase three days after elevated mean temperatures, confirming the lagged effect of exposure to hot temperatures observed in other studies.
Turcotte Tremblay 2024	Long term	45 362 respondents across 113 schools (47% male, 11% under the age of 12 years).	This study found that 9% of adolescents have trouble sleeping related to climate anxiety and that 6% have interference with work at least sometimes. This study shows that, compared to male adolescents, those who do not identify as either male or female are significantly more likely to experience impairments, to believe they can contribute and to try to change their behaviour. The researchers found that impairment due to climate anxiety was higher among adolescents from less affluent families.
Turmel 2023	Short term	40 participants from Quebec including health professionals who have experienced heat waves, mental health and well-being organizations, risk management organizations and citizens living in at-risk areas including older adults and individuals with mental health issues.	This research found that heat waves have significant psychosocial impacts, including increased anxiety and stress, social isolation, emotional distress and fatigue and exhaustion. The populations most at risk include older adults, people with mental health conditions, socially isolated individuals and people with low income or reduced mobility.
Valois 2020	Short term	Adults who live in flood-prone zones in Quebec surveyed in 2015 (1951 respondents; mean age = 57.3 years, 44.5% male) and 2019 (974 respondents; mean age = 60.7 years, 40.7% male).	In 2015, 25.5% of respondents reported mental health problems. In 2019, 35.4% of respondents reported mental health problems. This was not a statistically significant difference between years.
Vamvalis 2023	Long term	Interviews with three youth activists, three focus groups with youth and educators and interviews with six educators.	When asked how the climate crisis impacts participant and peer well-being, the youth activists involved in this study were unanimous in asserting the profound deleterious impacts of climate anxiety, dread and grief on youth mental health. While the participants struggled with overwhelming and complex feelings, they acknowledged that working collaboratively with others around a shared vision for transformative change positively affected their mental health. Collaborating for radical change acted as a counterpoint and balm to their anxiety and despair.
Vergunst 2024	Long term	1325 individuals born in 1997–1998 in Quebec (Indigenous communities and very remote regions excluded). 80.1% White. Reported at 23 years of age.	Most participants were worried about climate change with more than four-in-five reporting being at least “somewhat worried.” In the first analysis, participants who were worried about climate change had significantly higher concurrent symptoms of anxiety, depression and self-harm behaviours (including clinically significant levels of depression and self-harm). These associations were partially explained by mental health symptoms reported six years earlier. Further analyses showed that adolescents with higher anxiety symptoms at age 15 and 17 years were significantly more likely to worry about climate change at age 23 years, while adolescents with higher aggression-opposition symptoms were significantly less likely to worry about it, even after adjustment for contemporaneous anxiety symptoms. Adolescent inattention-hyperactivity and depression symptoms were not significantly associated with subsequent worry about climate change. The results underscore the need for caution when interpreting cross-sectional associations between climate worry and psychological distress without adjusting for prior mental health symptoms.
Verstraeten 2021	Intermediate term	200 women (27% pregnant, 73% postpartum). Mean maternal age at delivery = 30.9 (SD = 3.8). 96% in a relationship. Approximately 85% of the subjects had high socioeconomic status (family annual income of $100 000+).	High levels of social support satisfaction, but not the number of people, had a significant moderating effect on the relationship between peritraumatic distress and PTSD-like symptoms. There was no moderating effect of resilience.
Vida 2012	Short term	Individuals aged 15 and older in Quebec who visited the emergency department between 1995 and 2007.	Higher temperature and humidity were associated with statistically significant and primarily monotonic increases in emergency department visits for mental and psychosocial problems in three meteorologically homogeneous areas of Quebec. For temperature, significant increases were seen both for people under age 65 and for those aged 65 and older in two geographic areas; in one area the increases were seen only for those under 65. For humidity, significant increases at one or both higher humidity levels were seen for those under age 65 in all three geographic areas.
Villeneuve 2023	Short term	50 800 suicide deaths identified across Canada from 1 January 2002 to 31 December 2015.	This study found positive associations between daily increases of O_3_, NO_2_, fine particulate matter (PM2.5) and temperature and death from suicide. While positive associations were observed across different exposure lags, the strongest estimate was observed with a 3-day moving average for all pollutants. In contrast, for temperature, the strongest association was found with the same day average. The associations between increases in NO_2_, PM2.5, and temperature and suicide deaths were higher among females. Furthermore, the associations for each pollutant and temperature were stronger during the warm season.
Wang 2014	Short term	Residents of Toronto from 1 April 2002 to 31 March 2010.	For the residents of Toronto, Canada, exposure to high ambient mean temperature is significantly associated with increases in hospital emergency room visits for individuals with mental and behaviour disorders. The greatest effect/risk of emergency room admissions is limited between 0 and 4 lag days for hot temperatures. Age group–stratified analysis revealed that the effect of high temperature on MBD-related ER visits was greatest among individuals aged 60+ and 0–14. No significant differences were seen between genders and no significant cold associations were observed. With respect to specific mental illnesses, significant increases in hospital emergency room admissions during high temperatures were shown for schizophrenia and schizotypal disorders, mood disorders, substance abuse–related mental and behaviour disorders and neurotic disorders.
Woodhall-Melnik 2019	Long term	Interviews with key informants (local community leaders and those with inside knowledge on disaster relief efforts). Focus groups with 20 adult residents of southern New Brunswick approximately 1 year after the flood.	Negative mental health impacts following flooding include exhaustion, stress, anxiety, worry and uncertainty.
Yoon 2024	Long term	35 adults with schizophrenia who were living in British Columbia during the 2021 heat dome. 66% male; mean age = 41 years (SD = 17 years); 74% single.	Psychologically, the heat led to increased anxiety, irritability and disrupted sleep patterns, which further compromised mental stability and daily functioning. These emotional responses, coupled with cognitive fatigue, underscore the psychological burden imposed by environmental stressors. Many of the participants also shared that they regularly engaged in substance use at the time of the 2021 heat dome and wove together stories of being under the influence of drugs or alcohol while experiencing heat stress. The intersection of psychosis, substance use and heat stress symptoms presents a complex challenge.

**Abbreviations:** BRS, brief resilience scale; CLSA, Canadian Longitudinal Study on Aging; DSM-5, Diagnostic and Statistical Manual of Mental Disorders, 5th Edition; ED, emergency department; ER, emergency room; GAD, generalized anxiety disorder; GAD-7, Patient Health Questionnaire Generalized Anxiety Disorder Scale; GHQ, General Health Questionnaire; HA, hospital admission; IQR, interquartile range; IRR, incidence rate ratio; MBD, mental and behavioural disorder; MDD, major depressive disorder; OR, odds ratio; PCL-C, PTSD Checklist for DSM-5, civilian version; PHQ, Patient Health Questionnaire; PRR, prevalence rate ratio; PTSD, posttraumatic stress disorder; SD, standard deviation; WHO-5, World Health Organization-5 Well-being Index.

### Article characteristics

Only one study was published before 2000,^13^ six studies were published between 2000 and 2010, 13 studies were published between 2011 and 2018, and 41 studies were published between 2019 and 2024. Most studies were conducted in Alberta (n = 30; 42%), and 21% (n = 15) of studies were conducted in Quebec (see Figure 2). The climate and weather events and exposures included wildfire, flooding, perceptions of general climate change, extreme temperatures, and air pollution. Studies that involved youth under the age of 18 years accounted for 24% of the articles. Most articles included used quantitative questionnaires (57%), while 25% were qualitative research, 4% involved mixed methods, and 13% used health records for quantitative results. Qualitative information was used to inform the factors associated with mental health outcomes (Figure 3).

**Figure 2:**
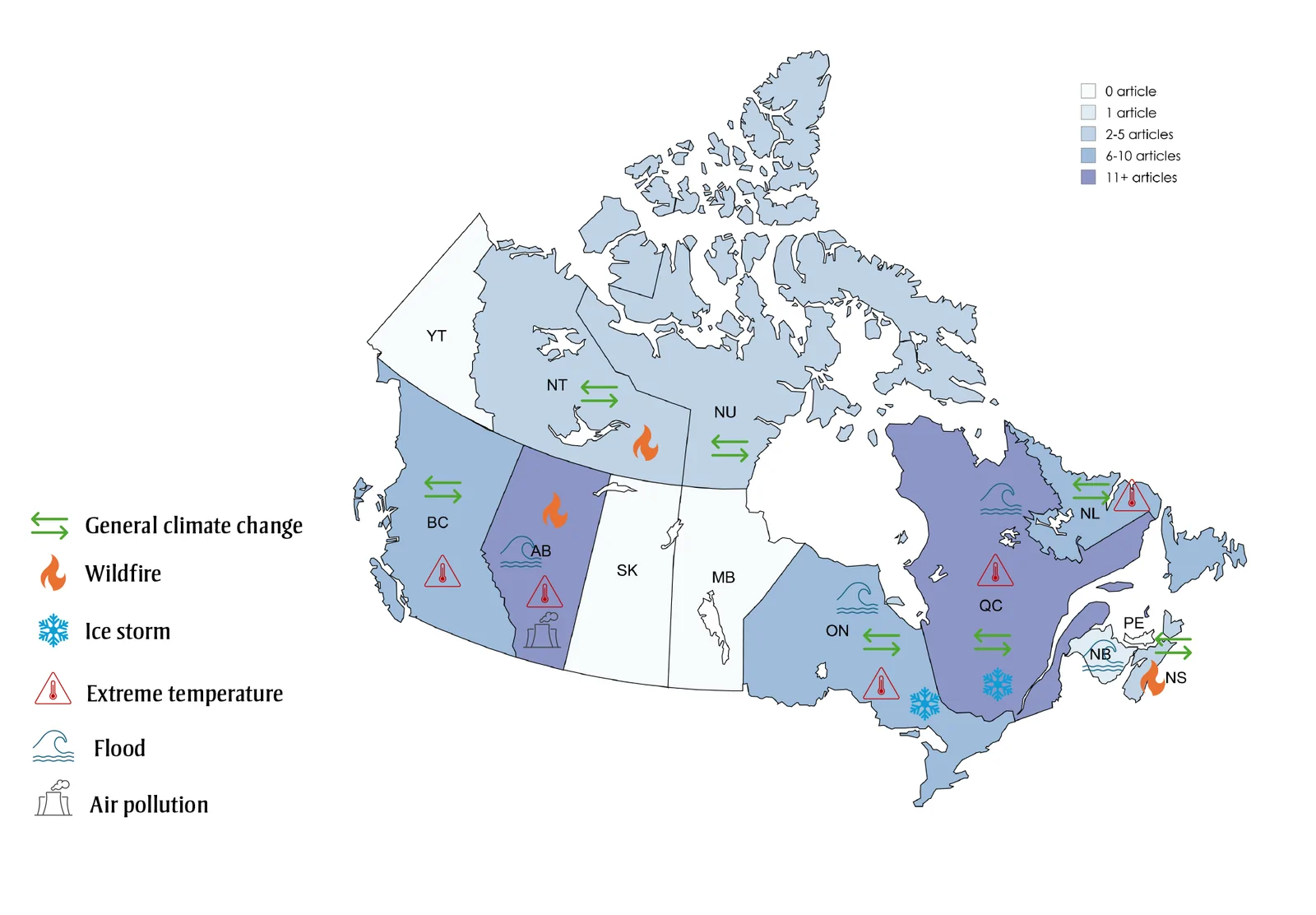
Geographic representation of province of focus and type of climate event across articles **Abbreviations:** AB, Alberta; BC, British Columbia; MB, Manitoba; NB, New Brunswick; NL, Newfoundland and Labrador; NS, Nova Scotia; NT, Northwest Territories; NU, Nunavut; ON, Ontario; PE, Prince Edward Island; QC, Quebec; SK, Saskatchewan; YT, Yukon. **Note:** Two articles are not included as they encompassed all of Canada. These articles included general climate change, extreme temperature and air pollution.

**Figure 3:**
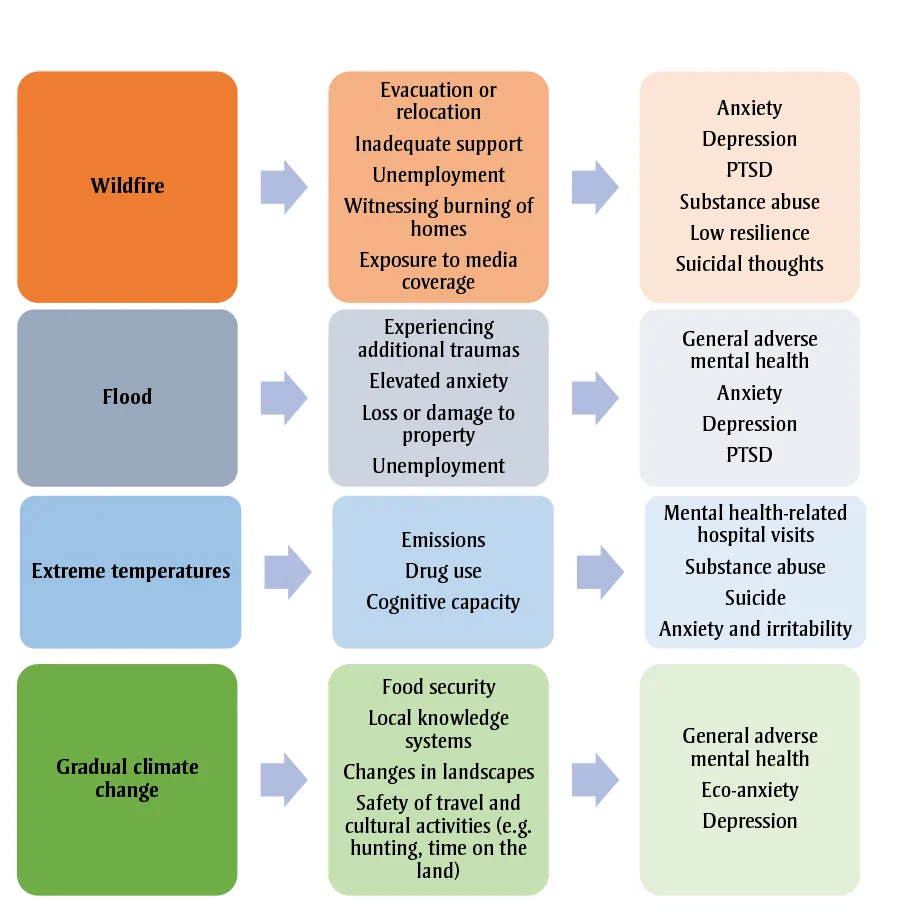
Potential factors influencing the impact of climate events on mental health outcomes **Abbreviation:** PTSD, posttraumatic stress disorder.

### Settings and climate events

These studies were conducted in community, hospital, clinic, and school settings. Studies included extreme weather and climate events (e.g. wildfires, extreme heat), gradual onset climate changes (e.g. sea level rise), and generally perceived climate change were reported in the literature. Extreme weather and climate events such as wildfires and floods commonly reported outcomes such as depression (n = 22 studies), anxiety (n = 23 studies), and PTSD (n = 18 studies). Generally perceived changes in climate included weather and seasonal patterns, and perceived changes in the spatiotemporal distribution of vegetation and wildlife. These studies were largely qualitative in nature, were focussed on themes such as general adverse mental health (n = 14 studies), eco-anxiety (n = 8 studies), and depression or grief (n = 7 studies).

### Reported burden of climate change–associated mental health outcomes

Reported rates of depression after exposure to extreme events and/or general climate change ranged from 5.2%^14^ to 53.7% (mild to severe depression).^15^ This included any mention of depression; depressive symptoms; mild, moderate, or severe depression; and probable or diagnosed major depressive disorder. Prevalence of anxiety ranged from 12.5% (anxiety symptoms;^14^ to 63% (climate anxiety)^16^ which included definitions such as anxiety, likely anxiety, high anxiety, other anxiety disorders, moderate to severe anxiety, and probable or confirmed diagnosis of generalized anxiety disorder (GAD). PTSD prevalence ranged from 10.2% (likely PTSD;^17^ to 60% (significant PTSD symptoms).^18^ The reports of PTSD included likely PTSD, elevated PTSD, provisional PTSD diagnosis, and presence of PTSD. The prevalence of substance (alcohol or drugs) use ranged from 7.9% (substance use disorder)^19^ to 17% (“substance abuse” in 2018).^20^ The definitions in the included articles were presence of alcohol use, self-reported increase in alcohol or drug consumption, high-risk and harmful drinking, probable alcohol/substance use disorder, alcohol use disorder, drug-related problems. The other mental health outcomes identified through the review were: distress, low resilience, insomnia, somatoform disorder, bulimia, binge eating disorder, panic disorder, thoughts of suicide and self-harm, psychotic disorders, and other mood disorders Table 1). Twenty-three studies reported on short-term impacts on mental health, 10 studies reported on intermediate-term effects, and 46 studies reported on long-term impacts on mental health. Anxiety was the most reported short-term outcome (n = 10 studies), PTSD was the most reported intermediate-term outcome (n = 7 studies), and anxiety was the most reported long-term outcome (n = 25 studies) (see Figure 4).

**Figure 4:**
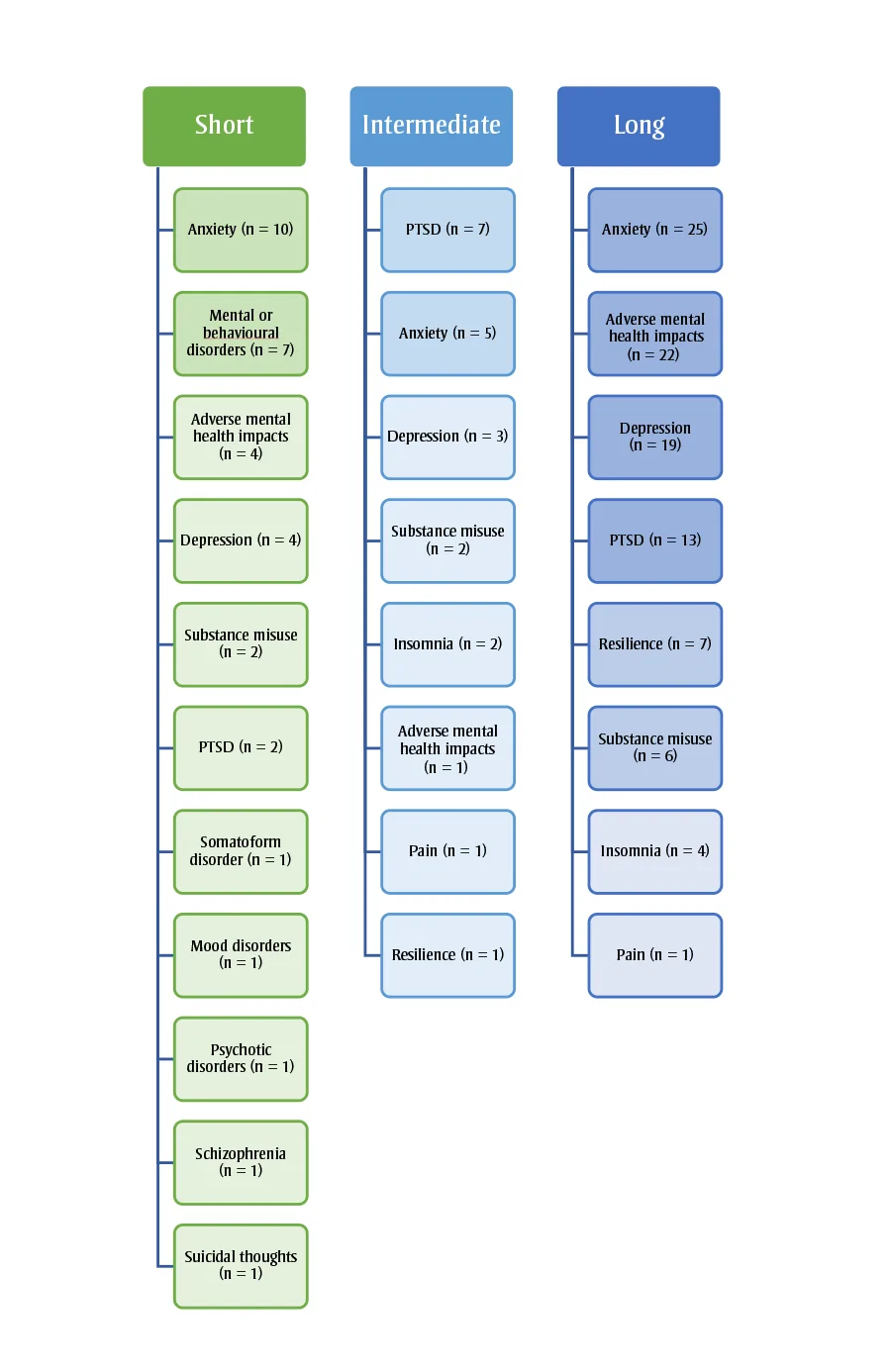
Outcomes related to time since climate event **Abbreviation:** PTSD, posttraumatic stress disorder.

### Climate event–associated exposure factors

Factors that influence the impact of climate and weather events on mental health outcomes were created by assessing common themes across the selected articles. The most reported exposures after a weather or climate event were evacuation, destruction of home, social and welfare issues, impacts on livelihood/cultural activities, and re-traumatization. These factors were found to be most relevant to Canadian climate change events and are likely to contribute to or exacerbate mental health conditions. The most frequent mental health outcomes following these exposures were anxiety, PTSD, depression, substance use, and low resilience (Figure 3). Factors that are specific to certain weather and climate events were also summarized in Figure 3. The exposure factors for specific weather and climate events and gradual environmental changes are presented below.

### Wildfires

Wildfires were the most frequently examined extreme weather or climate exposure, although most of these studies reported on the Fort McMurray area in Alberta. These findings identified multiple at-risk population subgroups such as those with a diagnosed mental illness,^21^^-^^25^ school-aged youth,^20^^,^^24^^-^^28^ women,^29^^-^^33^ and pregnant and postpartum individuals.^31^ The exposure factors following a wildfire event involved witnessing wildfires burning structures,^22^ exposure of media coverage,^32^ unemployment,^21^^,^^34^ evacuation or relocation,^32^ and receiving inadequate support from the government, family, or friends.^22^^,^^32^^,^^33^ Anxiety (n = 15 studies), depression (n = 15 studies), PTSD (n = 12 studies), “substance abuse” (n = 7 studies), resilience (n = 4 studies) and suicidal thoughts (n = 4 studies) were among the most reported mental health outcomes through these factors (Figure 3).

### Flooding

At-risk groups include women and individuals with mental illness.^14^ The potential factors influencing mental health outcomes include experiencing additional traumas like COVID-19,^35^ individuals with pre-existing elevated anxiety,^14^ experiencing loss or damage to property,^14^ and unemployment.^15^ Mental health outcomes were similar to studies investigating wildfires, which were general adverse mental health (n = 7 studies), anxiety (n = 6 studies), depression (n = 6 studies), and PTSD (n = 6 studies) (Figure 3).

### Extreme heat

Individuals at a particularly high risk for poor mental health outcomes due to extreme temperatures include women,^36^ older adults,^37^ youth,^37^ individuals with dementia,^38^ schizophrenia,^37^^-^^40^ mood disorders,^37^^,^^38^^,^^40^^,^^41^ immigrants,^42^ individuals with greater social or material deprivation,^38^ individuals with substance use disorder,^37^^,^^38^^,^^40^ and individuals with pre-existing health conditions.^38^ The factors that may influence mental health outcomes include cognitive capacity,^41^ emissions^36^^,^^42^ and drug use^43^ (Figure 3). Resulting mental health outcomes included mental health–related hospital visits (n = 5 studies), “substance abuse” (n = 2 studies), suicide (n = 1 study), and anxiety (n = 1 study) (see Figure 3).

### Gradual changes in weather and climate patterns

At-risk groups include Indigenous people,^44^^-^^46^ individuals with anxiety,^47^ women and gender-diverse people,^48^^-^^50^ individuals with limited access to education^44^ and health services,^51^ and those who lack financial security.^44^^,^^48^ Several studies discussed subjective reports of gradual changes in climate and weather patterns. Individuals from these studies were largely impacted by exposures to changes in food security,^49^^,^^52^ local knowledge systems,^49^ landscapes,^53^^,^^54^ and impacts on the safety of travel and cultural activities^44^^,^^45^^,^^49^^,^^53^^,^^55^^,^^56^. These factors led to commonly reported mental health outcomes of general adverse mental health (n = 14 studies), eco-anxiety (n = 8 studies), and depression (n = 7 studies) (Figure 3).

## Health equity information

The articles highlighted a number of at-risk populations such as: Indigenous communities (n = 16 studies),^44^^,^^49^^,^^56^ adolescents and youth (n = 17 studies),^20^^,^^57^^,^^58^ women and gender-diverse people (n = 12 studies),^33^^,^^36^^,^^59^ and individuals with pre-existing mental illnesses (n = 14 studies).^21^^,^^34^^,^^37^

### Indigenous peoples

While the studies including Indigenous populations provided rich qualitative information, they lack quantitative information on mental health outcomes associated with climate change. The qualitative data extracted from these studies demonstrated concerns for impacts on activities required for sustenance, which were also intertwined with cultural identity and livelihood of Indigenous communities (e.g. caribou hunting and berry picking). Indigenous peoples who live off the land are particularly at risk to future climate change as food insecurity—an ongoing crisis—is likely to worsen.

### Youth

Qualitative studies demonstrate that youth are likely to experience climate anxiety and despair.^47^^,^^48^^,^^60^ Following wildfires, youth can experience PTSD, depression, “substance abuse”, and suicidal thinking.^24^^,^^25^

### Women and gender-diverse people

In addition, women are more vulnerable to the psychological impacts following climate events^14^^,^^30^ or due to the ongoing climate crisis.^59^^,^^61^ Two studies showed that gender-diverse people are more likely to experience eco-anxiety.^48^

### Individuals with pre-existing mental illnesses

Finally, individuals with pre-existing mental health conditions are at risk of exacerbating psychological symptoms such as depression, anxiety, and PTSD from varying climate events.^32^^,^^34^^,^^41^ Individuals with schizophrenia are particularly at-risk for mental health-related admissions to hospital and suicide during extreme heat.^37^

## Discussion

This scoping review highlights the growing body of peer-reviewed research examining the mental health impacts of climate change-related events in Canada, with a notable increase in publications over the past five years. We identified 72 studies that met the inclusion criteria, comprising a range of quantitative, qualitative, and mixed-methods designs. The evidence points to a wide spectrum of mental health outcomes associated with both extreme (e.g. wildfires, floods, extreme heat) and gradual (e.g. shifting weather patterns, environmental degradation) climate exposures. Commonly reported outcomes included anxiety, depression, and PTSD, with varying prevalence estimates across populations, regions, and events. The studies also identified key factors—such as displacement, loss of livelihood, and cultural disruption—as potential critical mediators of these mental health outcomes. Importantly, findings underscore the disproportionate burden of climate-related psychological distress among specific populations, including Indigenous communities, youth, women, gender-diverse individuals, and those with pre-existing mental health conditions.

A notable geographic limitation in the current literature is the disproportionate focus on Alberta, with 42% (n = 30) of included studies conducted in that province—many of which centred around the Fort McMurray wildfires.^22^^,^^31^^,^^32^ An over-representation of Fort McMurray studies appear from overlapping samples as it relates to youth cohorts and clinical populations that raise a concern about the generalizability of certain outcomes. Only three of the 30 studies in Alberta conducted population-based research, while the remaining focussed on a specific population subset or follow-ups. While this focus reflects the severity and visibility of wildfire events in Alberta, it highlights a gap in the evidence base for other regions of Canada, particularly the Prairie provinces of Saskatchewan and Manitoba, as well as the Atlantic provinces. No studies captured mental health impacts in the Prairies, despite increased threats of flooding, extreme heat, and shifts in ecosystems combined with the unique rural location.^10^ In Atlantic Canada, unique climate vulnerabilities such as flooding, sea level rise, and coastal erosion may affect mental health and warrants investigation. This lack of geographic diversity limits the generalizability of findings, emphasizing the need for region-specific research to inform equitable climate and mental health policy across the country.

The four primary climate event-associated exposures identified in this review—wildfires, flooding, extreme heat, and gradual environmental changes—are consistent with global evidence linking climate-related events to adverse mental health outcomes. In this review, we identified that wildfires precipitate elevated rates of anxiety, depression, PTSD, and substance use, particularly in the aftermath of large-scale disasters such as those in Fort McMurray,^24^^,^^31^^,^^32^ echoing international findings from Australia and the United States where fire-related trauma has led to prolonged psychological impacts.^62^^,^^63^ This review showed that flooding is associated with higher rates of depression, anxiety, and PTSD, which is similar to research in the United Kingdom, demonstrating greater impacts on females compared to males.^64^ The mental health consequences of extreme heat—such as increased psychiatric admissions, substance use, and suicide—have also been widely reported across North America and Europe,^37^^,^^38^^,^^40^^,^^65^ with socially and medically vulnerable populations at greatest risk. Finally, gradual climatic changes, including disrupted seasonal cycles and environmental degradation, disproportionately affect Indigenous communities whose cultural and physical well-being is closely tied to the land. This aligns with growing global recognition of the mental health toll of ecological grief and loss, particularly in Arctic and rural regions.^44^^,^^45^^,^^66^ These findings underscore the importance of policy responses that address both the short- and long-term mental health risks of climate change across diverse geographic and cultural contexts.

The prevalence of depression (7.5% population baseline vs. 5.2%–53.7%), PTSD (8.5% vs. 10.2%–60%), anxiety (5.2% vs. 12.5%–63%), and alcohol use disorder (2.2% vs. 7.9%–17%) was typically higher among individuals exposed to extreme weather and climate events or gradual environmental changes when compared to non-exposed national population estimates.^67^^,^^68^ These findings underscore the psychological toll of climate-related exposures, even within a high-income, well-resourced context like Canada. That said, these findings should be interpreted with caution given that few studies were population based. While it is well established that PTSD and other mental health disorders often emerge in low- and middle-income countries following disasters—such as in parts of South America and sub-Saharan Africa^69^^,^^70^—this review adds to the growing body of evidence that climate change poses serious mental health risks in highly developed nations as well. Notably, studies from the United States and Europe have also documented similar trends, where events like hurricanes, floods, and heatwaves have contributed to significant increases in psychological distress and psychiatric morbidity.^71^^,^^72^

The broad range in reported prevalence likely reflects differences in study populations, exposure intensity, and time elapsed since the climate event. This review highlights a lack of research aiming to determine causal relationships between climate events and mental health outcomes and instead focusses on prevalence estimates. Included studies were cross-sectional in design which limited the ability to attribute mental health outcomes to specific exposures. Longitudinal or quasi-experimental designs are required to further isolate climate effects. Further, most studies were published after 2019, demonstrating greater interest in this topic due to more visible impacts of climate change. Studies published before 2019 measured mental health differently than studies published later, as more studies evaluated non-pathological mental state indicators such as climate anxiety and grief.^50^^,^^58^ This suggests an evolving discourse around climate change and mental health, and consistent terminology and measurement may be needed in the future.

Dimensions related to equity were unfortunately reported only descriptively or inconsistently across studies. Sex and gender were most reported, suggesting differential mental health impacts after exposure, particularly among women.^30^^,^^36^^,^^44^^,^^49^^,^^52^^,^^59^^,^^61^ Other social determinants such as race/ethnicity were less frequently reported compared to Indigenous experience and socio-economic status. Evidence from the gathered studies emphasized the role of land-based practices, cultural continuity and colonial context in shaping mental health impacts while socio-economic status was often referenced in connection with housing instability, employment precarity and access to resources after a climate-related event.^44^^,^^46^^,^^53^^-^^55^ This gap highlights the scarcity of equity-related dimensions as it relates to climate-related impact on mental health. This is a limitation of the current study as well as the availability within the Canadian literature which requires further study.

This review also signals a need for research on early intervention and mental health support in disaster-affected communities. PTSD appears to be an increasingly common outcome following environmental disasters.^17^ As climate change accelerates, mental health interventions must evolve to prioritize both immediate psychological first aid and long-term resilience strategies, including community-based trauma-informed care, accessible counselling services, and culturally safe spaces and supports. Proactive investment in mental health infrastructure and disaster preparedness will be essential to mitigating the cumulative mental health burden of future climate-related events. Additionally, there is a need for targeted supports for equity-owed groups including tailored resources for individuals with schizophrenia during heat waves, women during evacuations or relocations, and climate crisis support groups for youth.

## Strengths and limitations

This review has several strengths and limitations. Firstly, we followed a systematic approach with regards to searching for articles, screening abstracts and full texts, and extracting data duplications. However, the diversity in study design, population, setting, exposure measures, outcomes, and location among the included studies made it difficult to conduct a meta-analysis. Further, we limited the search strategy to only include empirical primary research, which may have limited the inclusion of population-level assessments and post-event reports that may be published in the grey literature. Another limitation is that the content analysis of the factors that may impact mental health outcomes was conducted by one person (SVCL), who counted the number of times a certain exposure or mental health outcome was mentioned in the datasheet and included the top five variables that appeared the most often. Some of these ratings were subjective, as mentions of certain information such as socioeconomic impacts were broad in the datasheet. This could affect the findings due to the subjective nature of the method of interpretation. Similarly, most articles were cross-sectional in nature which limits the ability to understand causal relationships and has important implications for applying findings to policy and practice. Importantly, this analysis was not meant to be a causal analysis but rather provide a framework or hypotheses for understanding how climate events may impact mental health through various factors. A third limitation is that the information presented in this narrative review is heavily concentrated in Fort McMurray, Alberta, due to the area experiencing two climate disasters in the span of five years, which can impact generalizability. In addition, many of these studies used the same dataset, rather than collecting data and reporting results from different populations within Fort McMurray. This also points to the fact that there needs to be more studies conducted in other provinces.

## Future research

There are several avenues for future research. It would be of interest to investigate other subgroups of interest such as immigrants and refugees, visible minorities, and people with chronic illness or disabilities, as their needs and risks may differ from those of other populations and they were largely unaccounted for in this review. Understanding factors related to social vulnerability is an important area of investigation as human-generated emissions will continue to cause further extreme changes in climate and weather patterns. There is also lacking information on other climate change-related weather events such as rising sea levels and hurricanes which are expected to impact Canada in the future.^10^ In addition, there is a lack of information from the Prairies and Atlantic Canada, and greater research is needed on these specific regions. The findings here point to a gap in our current knowledge due to low reporting on certain climate-related weather events as well as low reporting on mental health outcomes throughout the nation in primary research. Future work can also conduct an environmental scan of organizational and institutional reports related to climate change and mental health, which was not covered in this scoping review.

## Conclusion

In conclusion, this scoping review provides a synthesis of primary research on the mental health impacts of climate change in Canada, revealing a growing but mixed evidence base. The findings clearly demonstrate that both extreme and gradual climate-related exposures are associated with a significant increase in psychological distress, particularly depression, anxiety, PTSD, and substance use. This review situates Canada within the broader global discourse, showing that even in high-income countries with robust health systems, climate change can exert profound mental health consequences. Despite this, the literature remains geographically skewed and methodologically limited, with a heavy concentration on Alberta and a lack of causal evidence. There is a need to systematically evaluate climate change impacts on mental health across Canada to address the diverse population, and identify at-risk populations, exposures, geographic region and culturally sensitive interventions to meet equity-driven health needs.

## Data Availability

For more information on our data or requesting our datasheet, please reach out to the corresponding author.
